# Mechanisms of T cell evasion by Epstein-Barr virus and implications for tumor survival

**DOI:** 10.3389/fimmu.2023.1289313

**Published:** 2023-12-21

**Authors:** D. G. Sausen, M. C. Poirier, L. M. Spiers, E. N. Smith

**Affiliations:** School of Medicine, Eastern Virginia Medical School, Norfolk, VA, United States

**Keywords:** EBV, T cell, immune evasion, malignancy, tumor survival, cancer immune evasion, EBV and cancer therapy

## Abstract

Epstein-Barr virus (EBV) is a prevalent oncogenic virus estimated to infect greater than 90% of the world’s population. Following initial infection, it establishes latency in host B cells. EBV has developed a multitude of techniques to avoid detection by the host immune system and establish lifelong infection. T cells, as important contributors to cell-mediated immunity, make an attractive target for these immunoevasive strategies. Indeed, EBV has evolved numerous mechanisms to modulate T cell responses. For example, it can augment expression of programmed cell death ligand-1 (PD-L1), which inhibits T cell function, and downregulates the interferon response, which has a strong impact on T cell regulation. It also modulates interleukin secretion and can influence major histocompatibility complex (MHC) expression and presentation. In addition to facilitating persistent EBV infection, these immunoregulatory mechanisms have significant implications for evasion of the immune response by tumor cells. This review dissects the mechanisms through which EBV avoids detection by host T cells and discusses how these mechanisms play into tumor survival. It concludes with an overview of cancer treatments targeting T cells in the setting of EBV-associated malignancy.

## 1 Introduction

Epstein-Barr Virus (EBV) is a human oncogenic virus associated with various illnesses and malignancies (1, 2). EBV is widespread across all demographics, and the burden of chronic EBV infection has been estimated at greater than 90% of the global population (3). Classified as a gamma herpesvirus within the Herpesviridae family, EBV is also known as Human herpesvirus 4 (HHV-4). Its viral structure includes a tegument layer separating an inner viral capsid from an outer envelope containing surface glycoproteins that facilitate multiple viral functions including host cell entry, immune evasion, and viral assembly (4–6). Its double-stranded DNA genome, located inside the capsid, is estimated at 172 kbp (7). Primary EBV infection commonly occurs through the spread of saliva and infects oropharyngeal epithelial cells as well as B cells (8). It is typically asymptomatic in children (9), but often causes infectious mononucleosis (IM) in youth and adolescent populations who have not previously been infected (10). Colloquially known as “kissing disease” because of its spread through salivary transmission, IM is characterized by prolonged symptoms of fatigue, fever, cervical lymphadenopathy, pharyngitis, and splenomegaly. IM is usually self-limiting but can occasionally lead to severe illness (11, 12).

Primary acute EBV is followed by lifelong persistence in a latent state, which allows for reentry into the lytic cycle in life (13). EBV persistence in human host organisms has been associated with autoimmune conditions including multiple sclerosis (MS) (14), rheumatoid arthritis (RA) (15), and systemic lupus erythematosus (SLE) (16). The body of available clinical research demonstrates strong connections between EBV and malignancies such as nasopharyngeal carcinoma (NPC), gastric cancer (GC), and lymphoid cancers including Hodgkin Lymphoma (HL), Burkitt lymphoma (BL), diffuse large b cell lymphoma (DLBCL), NK/T cell lymphoma, and EBV-positive lymphoproliferative disorders (LPDs) such as post-transplant lymphoproliferative disorder (3, 17, 18). In 2010, EBV-associated cancers caused 1.8% of all cancer-related deaths worldwide, a number which had increased by 14.6% since 1990 (19). More recent data from 2020 indicate that the major EBV-associated malignancies, including NPC, GC, HL, BL, DLBCL, and NK/T cell lymphoma, are estimated to account for anywhere from 239,700-357,900 new cases and 137,900-208,700 deaths, indicating the extensive impact EBV has on global health (3).

EBV can escape recognition by the host’s immune system as well as actively prevent its own destruction by CD8+ cytotoxic T lymphocytes, which are crucial to controlling EBV infection (20, 21). Numerous viral products play multiple, varied roles in the immune evasion process, including glycoproteins (5), EBV-encoded RNA (22), and latent proteins (23). The virus’s capacity for immune evasion and disruption are usually insufficient to cause clinical concern in immunocompetent individuals but presents a serious risk of disease complications including development of EBV-associated malignancy in immunocompromised and immunosuppressed populations (24).

This review will begin with an overview of EBV infection, including both the lytic and latent cycles as well as latent protein expression. It will then review T cell evasion strategies of Epstein-Barr Virus and the consequent implications for tumor cell survival in infected individuals. It concludes with recent advances in treatments targeting T cells in EBV-associated malignancies.

## 2 Overview of infection

EBV primarily infects B cells and epithelial cells. Epithelial cells are typically the first to be infected by the virus (6, 25, 26). Entry into B cells is mediated by the formation of a heterotrimeric complex comprised of the glycoproteins (gp) gH, gL, and gp42, with human leukocyte antigen (HLA) class II molecules acting as receptors for gp42. gB acts as a viral fusogen (27).. Entry into epithelial cells is slightly different and requires only gH/gL and gB. gp42, which has higher degrees of expression in virus originating from epithelial cells, inhibits epithelial cell entry and thus dictates tropism (28). Gp350, a gp commonly targeted by neutralizing antibodies, is also important for entry into both B and epithelial cells (29). Once infection is established, the virus can enter lytic or latent phases of infection (30). Latent infection in B cells inhibits apoptosis and results in B cell transformation through the expression of a set of viral latency genes that allow for persistence over time in a quiescent state (31, 32). The set of genes expressed varies depending on factors such as cell type, length of time since infection, and the extracellular environment (33), but is broadly categorized into latency 0, latency I, latency II, and latency III (34, 35). During this phase, cytotoxic immunity prevents entry into the lytic cycle (36). Lytic reactivation may occur under several circumstances It may be initiated by cross-linking the B cell receptor with anti-immunoglobulin (37), and other factors such as immunosuppression and psychological stress can contribute to reactivation (13). Lytic infection involves the production of infectious virions (38). It is associated with much more robust gene expression than the latent cycle, including immediate early, early, and late genes (39, 40). The lytic state is much less common than the latent state; only 1 x 10^−4^ to 10^−5^ cells infected with EBV complete the lytic cycle and release mature virions (41).

### 2.1 EBV lytic phase

Lytic infection revolves around amplification of the viral genome and creation of structural proteins as well as viral capsids. Such structural proteins and expansion of viral elements is key for infecting other cells (42). Transition from latency to the lytic state is accomplished via the two transcription factors ZEBRA, also known as BZLF1 or Zta, and Rta, also known as BRLF1 (43). ZEBRA is particularly important as the master regulator of entry into the lytic cycle; indeed, expression of the ZEBRA protein alone results in a successful lytic cycle (44). ZEBRA binds to methylated regions of lytic DNA during latency to activate transcription of the repressed lytic genes, allowing the lytic phase to begin (45). The role of ZEBRA in EBV associated malignancies has not yet been fully elucidated, but it has been noted in Hodgkin’s lymphoma, diffuse large B cell lymphoma, and Burkitt lymphoma (46–48). Rta is essential for lytic DNA replication, binding to the origin of lytic replication (oriLyt) and potentially acting as a scaffold to recruit or assemble other proteins at the origin of replication (49). Notably, oriLyt is the area of the genome that mediates lytic phase DNA duplication (50). ZEBRA and Rta play key roles in manipulating protein expression profiles, altering cell cycle regulation, and promoting G0/G1 to S phase transition in host cells (51). Importantly, these two proteins induce each other’s expression (52).

Herpesvirus genes are expressed in three phases: immediate-early (IE), early (E), and late (L). Viral IE genes act to initiate transcription when the virus enters the host. IE gene products stimulate the expression of E and L genes or regulate the host to initiate virus replication. Many E genes are involved in DNA replication and many L genes create proteins required for virus assembly and egress (53–55). The previously discussed genes, Rta and ZEBRA, are classified as EBV immediate early genes (IE); they allow for the duplication of the genome, activation of later gene products in the EBV life cycle, and transactivation (56, 57). E genes play a variety of roles in the lytic cycle. For example, BHRF1’s role in inhibiting apoptosis through BCL-2 homology has been explored (58). BMRF1, another E gene, is a DNA Polymerase Processivity Factor (59) that was recently shown to activate transcription and limit the DNA damage response via interactions with the nucleosome remodeling and deacetylation (NuRD) complex (60). BNLF2a is another E gene implicated in immune evasion through inhibition of peptide transporter associated with antigen processing (TAP) (61). Expression of late genes requires completion of viral genome replication (53). BcLF1 and BLLF1 are both late genes; BLLF1 encodes the glycoproteins 350 and 220 (62), while BcLF1 encodes the major capsid antigen (63). Gp 350 is the most heavily expressed gp on the EBV envelope and is required for B cell attachment as discussed previously (62). Late proteins predominantly include viral structural proteins such as capsid, tegument, and glycoproteins (64).

### 2.2 EBV latent phase

EBV primarily maintains latency in B cells *in vivo*, although T cells and natural killer cells may be latently infected as well (65–67). The latent form of EBV infection is further categorized into four patterns, latency 0, I, II, and III, depending on the viral latent gene expression; the specific latent gene profile may also affect the reactivation rates later in infection (35, 68). During latency, EBV’s DNA can remain in episomal form or integrate into the host DNA, and replication of the viral genome is synchronized with the replication of chromosomal DNA during this latent phase (69–71).

EBNA1, 2, 3A, 3B, 3C, and LP are among the first latent genes expressed (33). Initial expression of latent membrane proteins (LMPs), such as LMP1, 2A, and 2B, begins during the first few days after B cell infection (72, 73). Increases in LMP1 expression are mediated at least in part by EBNA2 (74, 75). After initial B cell expansion, most latency genes are switched off and an EBNA1-dominant expression profile is induced (latency I). It is also possible that no EBV proteins are expressed, although EBV-encoded small RNAs (EBERs) may be present (latency 0). This has been proposed as a pertinent mechanism enabling EBV to evade detection by the host immune system, including CD8+ T cells (76).

### 2.3 Overview of EBV proteins expressed during latency

B cell EBV nuclear antigen 1 (EBNA1) is expressed in all latent phases except latency 0 (77, 78). It has important functions with regards to replication of the EBV plasmid genome. The C-terminus of EBNA1 binds to family of repeats (FR) and dyad symmetry (DS) sites of the latent viral origin of replication (oriP) (69). EBNA1 recruits the origin recognition complex (ORC) to DS and links the EBV plasmid to cell chromosomes at FR (79). Specifically, AT-rich hook DNA binding motifs allow EBNA1 to attach to cellular metaphase chromosomes (80). Tethering the EBV plasmid to the host chromosome is required for efficient replication (79). Recent research has indicated that EBNA1 is involved in processes beyond replication (81) such as immune evasion. For example, EBNA1 has been shown to reduce expression of the NK-cell receptor NKG2D ligands ULBP1 and ULBP5. When B cells were infected with null EBNA1 EBV, there was a significant increase in ULBP1 compared with wildtype-EBV, and similar results were obtained with ULBP5 when the EBNA1 binding site near the ULBP5 transcription start site was mutated (82). Moreover, these null EBNA1-infected B cells demonstrated increased susceptibility to NK cell-mediated killing and apoptosis. This function of EBNA1 contributes to immune evasion by reducing NK cell recognition of infected cells (82). In addition, EBNA1 contains a glycine alanine-rich sequence, and EBNA1 is only presented on MHC-I molecules when the glycine alanine-rich region is deleted, suggesting that this domain is involved in immune evasion by downregulating EBNA1 fragment presentation on MHC-I (83).

EBV nuclear antigen 2 (EBNA2) is expressed during latency III (78). As one of the first genes expressed during infection (84), it is essential in early latent gene reprogramming (85). EBNA2, along with the DNA binding protein CBF1, play significant roles as transcription factors for viral proteins by activating the viral promoters for EBNA1, LMP1, and LMP2A/B (86). It is required for efficient B cell transformation (87). In addition, EBNA2 has been implicated in oncogenesis. For example, it was shown to directly induce mRNA of the proto-oncogene MYC and cell adhesion molecules CD21 and CD23 (86). Mechanistically, it was recently shown that EBNA1’s α1-helix domain within the N-terminal dimerization (END) domain binds early B cell factor 1 (EBF1), a transcription factor that results in differentiation into B cell lineages. Cells infected by EBNA1 lacking the α1-helix domain were unable to progress past early S phase of the cell cycle, and there was decreased expression of MYC and MYC target genes (88). It has important functions regulating the immune response, for example by inducing expression of the IL-18 receptor. This has been directly attributed to the presence of EBNA2, as EBNA2-deficient EBV is unable to induce IL-18 receptor expression (89). IL-37 binding to the IL-18 receptor can induce an anti-inflammatory state (90), which could facilitate immune evasion during latency. Its role in T cell evasion will be discussed later.

EBV Nuclear Antigen 3 proteins A (EBNA3A), B (EBNA3B), and C (EBNA3C) are expressed during latency III (77, 78). There have been numerous studies using stop codons inserted into the open reading frames of EBNA3A, EBNA3B, and EBNA3C that have shown EBNA3A and EBNA3C are necessary for transformation of EBV-infected B cells (91, 92), although EBNA3B is not (93). EBNA3A and EBNA3C function as oncogenes by inhibiting tumor suppressor genes while EBNA3B acts as a tumor suppressor (94).

EBV Nuclear Antigen leader protein (EBNA-LP) is expressed during B cell latency III (77, 78). EBNA-LP primarily functions as an activator of EBNA2 in its activation of viral protein transcription (78). EBNA-LP has been implicated in contributing to the survival of EBV-transformed B cells, as infection by EBNA-LP knockout EBV plasmids caused death two weeks after infection in umbilical cord blood B cells B cell (95). Furthermore, it was found to enhance recruitment of transcription factors to the EBV genome (95).

Latent membrane protein 1 (LMP1) is expressed during latency II and III (13, 77, 78). LMP1 largely contributes to the proliferation of EBV-infected B cell lymphocytes. Low levels of LMP1 have been shown to severely inhibit B cell proliferation. Proliferative abilities were rescued once LMP1 was reintroduced (96). The proliferative effect of LMP1 in B cells is also seen following activation of the CD40 receptor without LMP1 present. This suggests that LMP1 provides the same proliferative effect on B cells as a constitutively active CD40 receptor (96). Signaling from the CD40 receptor is crucial for B cells to escape apoptosis in the germinal center (97). LMP1 contributes to oncogenesis via multiple mechanisms, including promotion of transformation, cell proliferation and survival, angiogenesis, and others besides (98). Its extensive role in oncogenesis can be explained by its impressive ability to manipulate cell signaling pathways, including NF-κB (99), EGFR (100), STAT (100), and JNK/AP-1 (101). In addition, LMP1 is associated with inhibition of T cell-mediated recognition of tumor cells. In hematologic malignancies, LMP1^+^ B cells are associated with higher expression of PD-L1 compared to LMP1^-^ B cells (102). Furthermore, LMP1 stimulates expression of indoleamine 2,3-dioxygenase 1 (IDO1), which inhibited B cell differentiation into antibody-secreting cells in germinal centers and indirectly suppressed the efficiency of neighboring B cells as well. In all, this resulted in impaired humoral immunity with potential implications for EBV immune evasion (103).

Latent membrane protein 2 (LMP2A/B) is expressed in latency II and III (77, 78). It is well known that the B cell receptor (BCR) maintains basal-level signaling in the absence of antigen recognition, that this is an important survival signal, and that it prevents induced apoptosis in the germinal center (104, 105). Previous studies have shown that BCR^-^ B cells can be saved from apoptosis with the introduction of LMP2A, and EBV-transformed, BCR+ B cells undergo apoptosis in an vitro model with low BCR expression following the removal of LMP2A. This suggests that LMP2A supports the survival and proliferation of B cells with low levels of BCR expression by constitutively mimicking BCR signaling and activating similar downstream pathways. This allows EBV-infected B cells with low BCR expression to evade apoptosis, contributing to EBV’s latency (106). Interestingly, it has been shown that LMP2A is not necessary for transformation of EBV-infected B cells that are able to avoid BCR downregulation and thus maintain high BCR expression (106).

LMP2A’s role extends well beyond BCR signaling mimicry. It generates significant transcriptional changes in the host cell impacting processes such as apoptosis, cell cycle progression, proliferation, and survival (107). LMP2A also contributes to EBV’s immune evasion by reducing antigen presentation on MHC-II. This is achieved by the downregulation of MHC-II receptors via the regulator class II transactivator (CIITA), which was shown to be largely driven by the immunoreceptor tyrosine-based activation motif (ITAM) on LMP2A (108). The ITAM motif of LMP2A has also been implicated in the inhibition of BCR-mediated signal transduction (109), which prevents induction of lytic replication (107).

**Figure 1:**
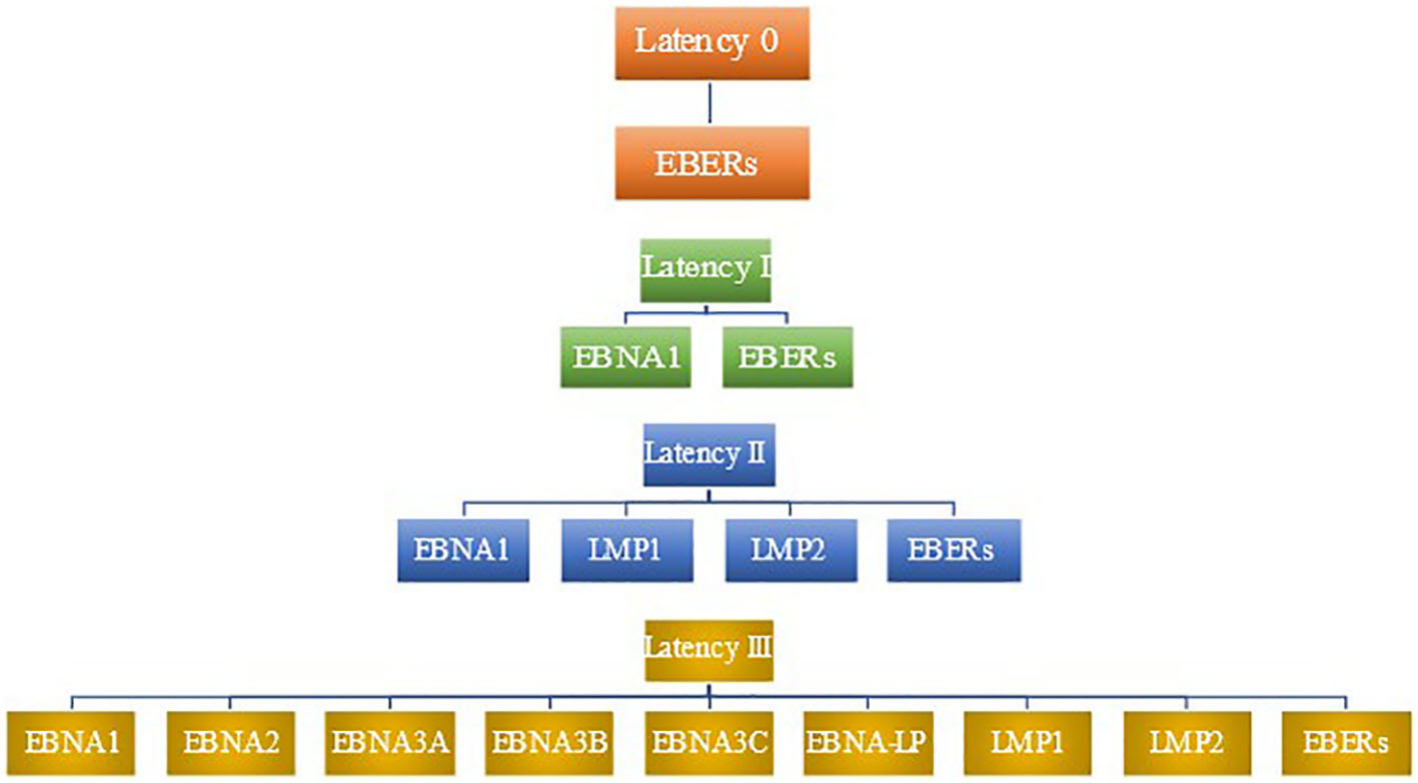
Patterns of latent gene expression in EBV. EBV expresses four distinct patterns of gene expression during latency, termed latency 0, latency I, latency II, and latency III. No proteins are expressed in latency 0, although EBERs are present (78). Latency I features the expression of EBNA1 as well as EBERs. In addition to the viral products seen in latency I, latency II includes the expression of LMP1 and LMP2. latency III has the most robust expression of gene products, including EBERs, EBNA1, EBNA2, EBNA3A, EBNA3B, EBNA3C, EBNA-LP, LMP1, and LMP2.

LMP2B is a smaller protein that has been shown to modulate the function of LMP2A (110). It has been implicated in sensitizing EBV-infected cells to signals promoting lytic induction. Rechsteiner et al. demonstrated that LMP2B overexpression increased the rate of lytic induction and decreased the threshold for lytic induction following BCR cross-linking. This opposes LMP2A’s role in preventing entry into the lytic cycle (111). **Figure 1** summarizes patterns of latent gene expression in EBV.

## 3 T cell response to EBV infection

The human adaptive immune response is partly comprised of T cells, which are lymphocytes that mature in the thymus and differentiate into CD4+ or CD8+ effector cells following antigen presentation. The former coordinate the immune response through the secretion of immunoactive substances such as cytokines, while the latter primarily function via cytotoxic mechanisms in which they induce target cell apoptosis (112). Different types of T cells recognize different types of MHCs and perform different functions (113, 114). For example, MHC class I-bound antigens are recognized by CD8+ cytotoxic T lymphocytes (CTLs) (115). CD8+ CTLs eliminate the infected cell through a variety of mechanisms, such as by infusion of granzyme/perforin or expression of cytokines like interferon (IFN) γ or tumor necrosis factor (TNF) (116). MHC class II-bound antigens are recognized by CD4+ helper T cells (115), which coordinate further immune response through B cell activation, T cell activation, activation of innate immune cells, and cytokine release (117). Naïve CD4+ T cells can be further activated into different types of helper T (Th) cells which have varying functions and immune properties (117). However, the distinction in function between CD4+ and CD8+ T cells is not absolute as EBV-specific CD4+ T cells have been shown to produce the cytotoxic proteins perforin and granzyme B (118). This functionality has been proven *ex vivo* in response to EBV-infected B cells, indicating a possible immune property that could be further explored in the development of an EBV vaccine (119). Other research into development of an EBV vaccine has determined specific eligible immunogenic T cell epitopes such as BXLF1 and BMRF2 that correlate with CD8+ T cell activation (120). Interestingly, a broader repertoire of EBV-specific T cell receptor (TCR) epitopes has been associated with MS. This may represent ongoing immune reaction to EBV (121), the importance of which is highlighted by the fact that cross-reactive antibodies between EBNA1 and the glial neuroadhesion molecule GlialCAM have been discovered (122).

High titers of EBV-specific CD8+ CTLs have been found in samples from IM patients, both in lymphoid cells (specifically Waldeyer’s Ring) and peripheral circulation (123). Effective control of acute EBV infection requires interactions between CD27 and CD70. Inhibiting this interaction in a mouse model resulted in uncontrolled EBV infection (124). It is worth nothing that the CD8+ T cell response to EBV has been postulated to relate to the pathogenesis and development of symptomatic IM itself (125). Persistent EBV infection has been shown to be controlled by the human adaptive immune response, particularly by CD8+ EBV-specific CTLs (126).These EBV antigen-specific CD8+ responses emerge during the first year following infection (127). Cytotoxic CD8+ responses predominantly target viral IE gene products of the lytic cycle as well as a subset of E genes. L proteins only rarely elicit a response. This is consistent with decreasing epitope presentation efficiency as the lytic replication cycle progresses (128). One potential explanation for the predilection of CD8+ T cells to favor IE protein targets is the expression of immunoevasins such as BNLF2a, which inhibits CD8+ T cell recognition, beginning during the early phase of the lytic cycle (129). Additionally, stronger responses were noted during acute infection (130). CD8+ activity against latent EBV infection focuses on EBNA3 family proteins (131).

Like CD8+ T cells, the CD4+ T cell response emerges within the first year of infection (127). The CD4+ response to EBV is less robust than the CD8+ response (21), but it is more varied (21) and more balanced across the stages of lytic infection (132). CD4+ T cells respond consistently to lytic cycle proteins but respond only rarely to latent cycle proteins (133). For example, a CD4+ T cell response against EBNA1 is present in many individuals with latent EBV infection (134). However, the ability of EBNA1 to localize in the host nucleus allows it to escape detection by the host immune surveillance system, specifically by minimizing its exposure to the host macroautophagy pathway. This in turn limits its CD4 epitope presentation (135). Notably, EBNA1 is the most immunodominant latent epitope targeted by CD4+ T helper 1 (Th1) cells, followed by EBNA3C. Much weaker responses were observed to LMP1 and LMP2 (136).

CD4+ T cells have demonstrated cytotoxic ability against lymphoblastoid cell lines (LCLs) (132). Indeed, primary infection results in the oligoclonal expansion of a set of Th1-like, EBV-specific CD4+ T cells. Contained within these CD4+ cells are cytotoxic proteins capable of delivering a swift response to ex vivo challenge (119). These cytotoxic CD4+ T cells can be maintained, as CD4+ T cells specific for the BORF1 capsid protein continued to express perforin and granzyme-B even during persistent infection (118). In summary, CD4 and CD8+ T cells work in tandem to combat EBV, with the CD8+ response being more heavily implicated in controlling infection. EBV is extremely effective at evading the immune response and establishing persistent infection in human hosts.

## 4 Modulation of T cell response and implications for cancer cell evasion

As mentioned above, EBV is associated with a host of cancers, including both hematologic (78) and epithelial (137) malignancies. It has developed numerous mechanisms to evade T cell responses, and some of these mechanisms have been implicated in oncogenesis, i.e. through immune modulation by upregulating PD-L1 (138) and inhibiting immune cell recognition (22).

### 4.1 Manipulation of the PD-1/PD-L1 axis

Programmed cell death ligand-1 (PD-L1) is an immune checkpoint inhibitor. Its corresponding receptor, programmed death ligand-1 (PD-1), is found on a variety of immune cells, including T cells. Binding of PD-L1 to its receptor dampens the immune response, for example by inhibiting the effector function of cytotoxic T cells (139) or converting CD4+ T cells, particularly memory CD4+ cells, to a highly inhibitory inducible Treg phenotype (140). This makes it an opportune target for both EBV and malignancy. Evidence supporting the idea that EBV infection can upregulate PD-L1 comes from experiments demonstrating that EBV-transformed LCLs express PD-L1. Indeed, the PD-L1 promoter and enhancer were both active in EBV+ LCL. The enhancer was noted to be AP-1 dependent. LMP1 was shown to upregulate both the AP-1 component C-jun and the PD-L1 promoter, with JAK3 representing a possible mechanism of PD-L1 promoter upregulation (141).

Other studies support the role of EBV latent proteins in altered PD-L1 expression (142). While EBV-infected B cells only expressed low levels of PD-L1 on infection day 0, there was a gradual increase in expression levels through day 28 post-infection with strong expression of PD-L1 by post-infection day 2. Infection with a mutant, EBNA2-deleted strain resulted in minimal PD-L1 expression. Infection with a LMP1-deleted strain yielded similar results, indicating both these proteins are important for early PD-L1 induction (142). In accordance with the paper by Anastasiadou et al. showing that EBNA2 decreased miR-34a expression and subsequently increased PD-L1 expression in B cell lymphomas (143), EBNA2 deletion resulted in a mild increase in miR-34a expression. Interestingly, LMP1 deletion resulted in a more significant, 3.1-fold increase in miR-34a, indicating LMP1 may also play a role in increased PD-L1 expression via miR-34a suppression (142). ChIPseq and ChIA-PET analysis demonstrated that EBNA2 binds the PD-L1 promoter region, and reporter assays demonstrated increased activity at PDL1Enh and PDL1Enh558 as well as a minor increase at PDL1Enh562. PDL1enh is in the PD-L1 promoter region, while the other two sites are structurally similar regions 130 kb and 170 kb downstream of PD-L1, respectively (142). Furthermore, EBNA2-induced induction of PD-L1 was recently documented in reactivated EBV across multiple cell lines. This induction was inhibited when tested in EBV+ cell lines where EBNA2 was knocked down (144).

### 4.2 Manipulation of the PD-1/PD-L1 axis in epithelial malignancies

Manipulation of PD-1/PD-L1 is heavily implicated in tumor immune evasion across multiple types of malignancies (145–147), and EBV-associated tumors are no exception. A study assessing the associations between EBV status and PD-L1 expression in nasopharyngeal cancer demonstrated a positive, statistically significant correlation between EBER positivity and PD-L1 expression (p = 0.004) (148). While this study did not demonstrate a statistically significant correlation between overall survival and PD-L1 or EBER expression (148), EBV levels and PD-L1 status have significant treatment implications (149), and other studies have indicated that higher EBV levels may be associated with poorer outcomes in patients receiving anti PD-1 therapy (150). EBV-associated gastric cancer (EBVaGC) likewise demonstrates higher levels of PD-L1 expression than its non-EBV associated counterparts (151, 152); A meta-analysis demonstrated that EBVaGC had increased PD-L1 expression in 54.6% of cases (152).

This upregulation is achieved through a variety of mechanisms. For example, Fang et al. demonstrated increased PD-L1 expression in EBV+ NPC cells. Both transfection and induction of LMP1 resulted in increased PD-L1 expression. Pathways implicated include JAK3/STAT3, MAPKs/AP-1, and NF-κB (153). LMP1 also interacts with the β-galactose-binding protein Lgals1 This allows it to impact the NF- kB pathway and subsequent IRF1 signaling, which results in higher PD-L1 expression. This effect is countered by the addition of the Lgals1 inhibitor OTX008. Lgals and PD-L1 expression were both correlated with higher rates of recurrence and metastasis than tumors that did not express these proteins (154).

Recent research has shown that LMP1 also induces soluble PD-L1. While the full significance of soluble PD-L1 requires further study, it acts as an immune inhibitor that can cause T cell impairment and apoptosis (155, 156). Furthermore, it is commonly expressed in malignancies (155). Consistent with prior results (153), LMP1 was shown to induce cellular PD-L1 in NPC cells. Furthermore, PD-L1 was detected in concentrated culture medium derived from LMP1-expressing NP69T cells consistent with the expression of soluble PD-L1. Soluble PD-L1 levels were increased in the serum of patients with NPC, with higher levels being associated with more advanced disease, although it was not associated with prognosis (157).

PD-L1 expression has been documented in multiple EBVaGC cell lines (158). While it was also demonstrated in lines not associated with EBV, only EBVaGC demonstrated increased PD-L1 expression when exposed to IFNγ. Importantly culturing Jurkat cells, a T cell lymphoma line, with NCC24 and YCCEL1 cells treated with IFN-γ increased the number of Jurkat cells arrested in G0/G1. This effect was not seen in the absence of IFN-γ and was partially abrogated by the addition of anti-PD-1 antibody. These results suggest that IFN-γ may play a role in PD-L1 expression and that this expression functionally impairs T cell proliferation in EBVaGC (158). IFN-γ has also been shown to act in conjunction with LMP1 to upregulate PD-L1 in NPC (153).

PD-L1 expression was positively correlated with expression of stimulator of interferon genes (STING), and expression of these molecules was associated with higher overall survival (OS) in EBVaGC (151). This contrasts with the findings of Yoon et al., who found that PD-L1 expression in EBVaGC promoted tumor proliferation, invasion, migration, survival, and immune escape with deleterious effects on prognosis (159). Likewise, Sasaki et al. found that PD-L1 played a key role in T cell evasion by EBVaGC (158). Nakayama et al. found that high EBV viral loads correlated with increased PD-L1 expression in EBVaGC and poorer prognoses (160).

miRNAs also play key roles in altering the PD-L1/PD-1 signaling. Working with gastric cancer and NPC cells, Wang et al. demonstrated that EBV-encoded miRNAs upregulate PD-L1 (161). One such example is miR-BART17-3p. Mechanistically, it was shown that transfecting this miRNA into EBV-negative cells resulted in PBRM1 inhibition, specifically by binding to the PBRM1 3′-UTR and causing mRNA degradation through the RNA-induced silencing complex (RISC). PBRM1 overexpression resulted in decreased PD-L1 expression. Conversely, PBRM1 inhibition resulted in increased PD-L1 expression (161). miR-BART11 was also implicated in PD-L1 upregulation by targeting FOXP1 (161). As was the case with miR-BART17-3p, mirBART-11-3p and mirBART11-5p both bound the 3′-UTR sequence of FOXP1 and lead to its degradation via RISC. Overexpression of FOXP1 lead to decreased PD-L1 expression, while FOXP1 inhibition led to increased PD-L1 expression. Furthermore, transfection of miR-BART11 and miR-BART17-3p into HONE1 and AGS cells resulted in increased T cell apoptosis and decreased tumor cell apoptosis (161). This is consistent with the idea that these miRNAs promote immune evasion through upregulation of PD-L1. miR-BART5-5p is another miRNA implicated in EBV-mediated PD-L1 upregulation, this time through the protein inhibitor of activated STAT3 (PIAS3)/STAT3 pathway (159). While PIAS3 mRNA levels were unaffected by miR-BART5-5p, protein levels were lower in gastric carcinoma cells transfected with miR-BART5-5p. Indeed, the 3’-UTR of PIAS3 was noted to have a potential binding site for miR-BART5-5p. This downregulation of PIAS3 resulted in increased activated STAT3 (pSTAT3) activity, which in turn increased PD-L1 activity (159).

### 4.3 Manipulation of the PD-1/PD-L1 axis in hematologic malignancies

PD-L1 manipulation secondary to EBV is also seen in hematologic malignancies (162), including BL and DLBCL (143). Like with other forms of cancer, PD-L1 expression has implications for prognosis and is associated with poor outcomes in multiple EBV-associated hematologic malignancies (163–165). The significance of this is highlighted by a study showing that 20% of sampled EBV-associated DLBCL demonstrated 9p24.1 amplification, which includes PD-L1 and PD-L2. Furthermore, 9 of 24 formalin-fixed and paraffin-embedded (FFPE) specimens demonstrated PD-L1 amplification, eight of which demonstrated strong amplification (166). In another study, 5/7 large B cell malignancies were associated with EBV positivity; these tumors demonstrated stronger PD-L1 than PD-1 staining (167). Interestingly, another study found that PD-L1 expression in EBV+ DLBCL was less common in Japanese patients (6/57 cases assessed), indicating ethnicity could potentially play a role in the frequency of PD-L1 expression in DLBCL, although differences in technique or cut off values were also postulated as an explanation (168). 35/90 NK/T cell lymphomas, all of which were associated with EBV, demonstrated PD-L1 staining. In classic Hodgkin lymphoma (cHL), 40/41 PD-L1 positive cases were EBV-associated (167), and 19/26 cases of EBV+ post-transplant lymphoproliferative disorder (PTLD) expressed PD-L1 (141). Working with NK cell lines NK-92 (EBV-negative) and SNK-6 (EBV-positive), Bi et al. demonstrated that PD-L1 expression was much higher in SNK-6 cells. PD-L1 expression was significantly higher in NK-92 cells transfected with LMP1, and LMP1 upregulated proteins involved in MAPK/NF-κB signaling. Furthermore, patients with increased PD-L1 expression, either soluble or within the tumor itself, had poorer response to treatment and lower survival rates; PD-L1 expression was found to be an independent adverse prognostic factor for patients with stage I to II NK T cell lymphomas (164).

miRNA also impacts PD-1/PD-L1 signaling in hematologic malignancies. Serum levels of the cellular miRNA (169) miR-155 were increased in DLBCL, with EBV-positive patients having higher levels than EBV-negative patients. miR-155 enhanced PD-L1 expression and caused CD8+ T cell apoptosis. CD8+ T cell effector function was inhibited via dephosphorylating AKT and ERK, and PD-L1 blockade inhibited tumors overexpressing miR-155 in murine xenograft models, implying PD-L1 inhibition promoted T cell effector function (170). **Table 1** provides an overview of mechanisms employed by EBV to upregulate PD-L1.

**Table 1:** Upregulation of PD-L1 by EBV.

Molecule	Mechanism	Cell/cancer line	Reference
PD-L1	Immune inhibitory molecule upregulated by EBV	Multiple	(139, 140, 145–147)
EBNA2	Inhibition of miR-34a	B cells	(142, 143)
EBNA2	Induction of PD-L1	Multiple cell lines	(144)
EBNA2	Promotes activity at PD-L1 enhancer	B cells	(142)
LMP1	Inhibition of miR-34a	B cells	(142)
LMP1	Alter signaling in multiple pathways (JAK3/STAT3, MAPKs/AP-1, and NF-κB) to upregulate PD-L1	NPC	(153)
LMP1	Interact with Lgals1 with subsequent impact on the NF- kB pathway and IRF1 signaling	NPC	(154)
LMP1	Induction of soluble PD-L1	NPC	(157)
LMP1	IFNγ	EBVaGC, NPC	(158, 171)
LMP1	Upregulates PD-L1 promoter through increased Jak3 and the PD-L1 enhancer through increased AP-1	LCL	(141)
LMP1	Upregulates MAPK/NF-κB signaling	Nk cells	(164)
miR-BART17-3p	PBRM1 inhibition	EBVaGC, NPC	(161)
miR-BART11	Target FOXP1 for degradation	EBVaGC, NPC	(161)
miR-BART5-5p	PIAS3 downregulation led to increased STAT3 activity	EBVaGC	(159)
miR-155	PD-L1 upregulation	DBLCL	(170)

### 4.4 Modulation of immunoactive cellular secretions – IFN

EBV’s involvement extends beyond the confines of the cell membrane; it is implicated in altering immunoactive cellular secretions as well (172–174). One such signaling molecule influenced by EBV is IFN. IFN signaling plays a significant role in T cell regulation through both direct and indirect signaling modalities (175). The impact can either be stimulatory or inhibitory depending on precisely when the T cell receptor is stimulated compared to type I IFN receptor (IFNAR) signaling (175). It is involved in CD4+ T cell expansion during acute infection and exhaustion during chronic infection (176), Treg polarization (177), CD8+ T cell proliferation and memory formation (178), and many other aspects besides (175, 176).

EBV has evolved numerous techniques for manipulating the IFN response, thus enhancing its immunoevasive capabilities (179). One protein that contributes to downregulation of the IFN response is the early protein BFRF1 (180). Cells transfected with BFRF1, the EBV protein BGLF4 (which was previously demonstrated to suppress IFN regulatory factor 3 [IRF3] signaling (181)), and vector were infected with sendai virus. BFRF1 inhibited IFN-β at a rate comparable to BGLF4. Consistent with these results, interferon-stimulated response element (ISRE) promoter activation was suppressed. Mechanistically, it was demonstrated that BFRF1 inhibited the kinase functionality of IKKi, which reduced IFN-β promoter activity. Subsequent IRF3 phosphorylation and dimerization was inhibited (180).

LMP2A and LMP2B also have deleterious effects on IFN signaling. Epithelial cells expressing LMP2A and 2B were less responsive to both IFN- α and IFN-γ. Transcriptional profiling indicated that these proteins had a global impact on IFN-associated gene expression, an effect mediated by increased degradation of IFN receptors (182).

The tegument protein BGLF2 has an inhibitory effect on IFN signaling (174). When cells transfected with BGLF2-expressing plasmids were exposed to IFN-β, there were reduced levels of phosphorylated Tyk2, phosphorylated STAT1, and phosphorylated STAT3 compared to cells transfected with plasmids expressing a BGLF2 carboxyl terminal deletion mutant or GFP. Similar decreases in phosphorylated STAT1 and phosphorylated STAT3 were noted following treatment with IFN-α in cells transfected with BGLF2-expressing plasmids but not with the BGLF2 mutant. Moreover, expression of the IFN-stimulated genes IRF1, IRF7, and MxA was decreased following treatment with IFN-α, although ISG15 expression was not. BGLF2 did not inhibit IFN-γ (174).

gp110 is critical in determining EBV tropism and in facilitating efficient infection (183). Recent research has examined its role in evading the IFN response (184). Like BFRF1 (180), it was shown to inhibit IFN-β promoter activity following transfection into human embryonic kidney 293T (HEK293T) cells. ISRE activity was inhibited, as was mRNA expression of IFN-β, ISG15, ISG56, IL-6, and IL-8. Furthermore, gp110 expression resulted in increased viral cytotoxicity and viral fluorescence following infection with herpes simplex virus 1-green fluorescent protein (HSV-1-GFP) or vesicular stomatitis virus-GFP (VSV-GFP) compared to vector. Vero cells, which do not produce type I IFN, had nearly no difference. This is consistent with the idea that gp110 functionally attenuates the host immune response (184). Mechanistically, it was shown that gp110 impairs IKKi kinase function by disrupting K63-linked polyubiquitination. In essence, binding of gp110 to IKKi blocks IKKi’s ubiquitination region, which impairs the ability of TRAF3 to regulate IKKi by polyubiquitination (184).

The same set of experiments demonstrated that gp110 affects β-catenin (184), a molecule known to stimulate IFN production (185, 186). β -catenin interacts with IRF3, which promotes p300 recruitment and IFNB1 promoter acetylation (186). Gp110 facilitates proteasomal degradation of β-catenin, which results in diminished IFN-β production. This inhibition was reversed following the addition of MG132, a proteosome inhibitor (184).

miRNAs play a significant role in modulating host IFN responses to EBV infection (187). Screening identified miR-BART1, miR-BART16, miR-BART22, and miR-BHRF1-2 as miRNAs that significantly decreased IFN levels as well numerous other miRNAs with a less significant but notable impact on IFN secretion. In addition, EBV miRNAs target other genes in the IFN signaling pathways as well as proteins involved in type I IFN secretion (187).

Consistent with the above results, Hookyaas et al. demonstrated that miR-BART16 plays a role in modulating the host IFN response. Indeed, when it was expressed in 293T-ISRE-mCherry reporter cells, ISRE reporter expression was decreased by 27%. Notably, cells expressing the miRNAs miR-BART1, 3, 4, 5, 6, 15, 16, 17, 18, and 21 decreased ISRE reporter expression by 52%, indicating other miR-BARTs may diminish the IFN response as well. Furthermore, induction of the interferon-stimulated genes IFIT1 and ISG15 was inhibited by miR-BART16. Mechanistically, it was shown that miR-BART16 targets the 3’UTR of CBP (188). miR-BART6-3p is another miR-BART that exerts a negative regulatory effect on IFN signaling, this time through retinoic acid-inducible gene I (RIG-I)-like receptor inhibition (189). RIG-I-like receptors sense viral infection and stimulate transcription of type I IFN and other antiviral genes (190). Not only did miR-BART6-3p inhibit host IFN-β response to EBV infection, it specifically targeted the 3’ UTR of RIG-I (189). In addition, RNA circBART2.2 decreases IFN-γ secretion by T cells via PD-L1 upregulation (191). IFN-γ secretion represents one mechanism by which T cells induce cytotoxicity in cancer cells (192).

In addition to the above modulation of IFN signaling, EBV-specific manipulation of the IFN pathway has been implicated in EBV-associated malignancy (171). The IFN response has multiple conflicting roles in cancer. On the one hand, it was recently shown that cancer-specific IFNAR 1 signaling resulted in immune cell exhaustion, release of cancer cell immune checkpoint receptor ligand-containing, cancer derived exosomes, and poor clinical outcomes (193). On the other hand, IFN signaling has been shown to augment granzyme B expression with subsequent augmentation in the cytotoxic capacity of T cells and subsequent suppression of tumor development (194). A more complete review of the role of IFN in cancer can be found elsewhere (195–197).

It has been proposed that EBV-associated cancers can be divided based on their manipulation of IFN genes (171). Ingenuity Pathway Analysis (IPA) demonstrated that upstream regulators of EBV response genes primarily involved type I or II IFN signaling, transcription factors that stimulate IFN, or molecules that overlap with the type I IFN response. Cancers with IFN upregulation including GC, NPC, and DLBCL comprised one group, while EBV-associated cancers with a diminished IFN response when comparing EBV-positive and EBV-negative cancers including Burkitt lymphoma, angioimmunoblastic T cell lymphoma, NK/T cell lymphoma, and sporadic Burkitt lymphoma comprised the other group (171). Cancers in the IFN+ group demonstrated increased expression of immune checkpoint proteins, including PD-L1 and indoleamine 2,3-dioxygenase (IDO)-1 (171). Indeed, IFNγ only induced IDO-1 in EBV+ GC cells, and EBV+ GC cells demonstrated increased induction of PD-L1 following IFN-γ treatment compared to their EBV-negative counterparts (171). This upregulation in PD-L1 is consistent with the previously discussed results obtained by Sasaki et al. demonstrating that IFN-γ enhanced PD-L1 expression in GC (158). Similar results have also been seen in NPC (153).

### 4.5 Modulation of immunoactive cellular secretions – interleukins and chemokines

Interleukins (IL) are signaling molecules expressed by several different cell types that stimulate multiple immune cell functions, including activation, differentiation, proliferation, migration, and adhesion (198). Like IFN, IL signaling plays a role in the immune response to cancer (199), and modulation of IL signaling by EBV thus has implications for both viral persistence and tumor cell survival. Il-1 is a pro-inflammatory cytokine that can stimulate T cell activation (200) and plays a role in stimulating the innate immune system (201). BHRF1-2 miRNAs have been shown to inhibit IL-1 signaling via inhibition of the IL-1 receptor 1. Indeed, IL-1 receptor 1 inhibition was noted at both the RNA and protein levels. In LCLBACD2 (a BHRF1-2 knockout cell line) and BJAB cells expressing BHRF1-2 miRNAs, levels of IL-1α and IL-1β transcripts were halved. Consistent with these results, secreted IL-1β was also halved in LCLBACD2 cells transfected with BHRF1-2 miRNA. Control LCLs had significantly higher levels of IL-1α, IL-1β, and IL-6 than miR-BHRF1-2-5p-sponged cells. The authors concluded that this interruption in signaling impacts autocrine/paracrine IL-1 signaling, which leads to alterations in the cellular environment and cytokine expression in latently infected cells (201). Interestingly, IL-1β has long been known to be upregulated in the setting of EBVaGC, where it may act as an autocrine growth factor (202).

T cell EBV-induced manipulation of IL-6 has been implicated in NPC. Immortalized nasopharyngeal cells infected with EBV demonstrated higher levels of phosphorylated STAT compared to their non-infected counterparts following IL-6 exposure. This effect is created through increased expression of the IL-6 receptor (203). IL-6 signaling increased invasive properties as well as expression of cyclin D1 and LMP1, whose oncogenic and immunoevasive capacities were discussed above. Transfection of a dominant active STAT mutant (STAT3C) promoted anchorage-independent growth and increased c-myc and Bcl-2 expression. Increased IL-6 receptor expression resulted in cell growth, including anchorage-independent growth (203). Collectively, these findings indicate that IL-6 receptor overexpression and subsequent increased IL-6 signaling increases cell growth and malignancy in EBV-infected cells. The significance of these findings is underscored by the fact that IL-6 receptor expression was detected in 62% of NPC samples (203).

IDO is a compound that has long been known to impair T cell-mediated immune responses (204). Stromal cell production of IDO was upregulated in the context of EBV-associated oral squamous cell carcinoma compared to EBV-negative cancers. Internalization of exosomes from P3HR1, an EBV-positive BL cell line, by monocyte-derived macrophages (MDM) resulted in expression of IDO, a result that was poorly replicated when using EBV-negative Akata cells. Mechanistically, it was shown that EBER-1 induced RIG-I expression, which led to increased TNF-α and IL-6 expression and subsequent upregulation of IDO. Importantly, increased IDO production by MDM impaired CD4+ and CD8+ T cell proliferation and inhibited CD8+ T cell cytotoxicity (205). This result is in accordance with previous experiments demonstrating that IL-6 and TNF-α resulted in increased IDO expression in macrophages associated with NPC. Notably, inhibition of p38/MAPK and NF-κB pathways nearly eliminated IDO expression (206). Both these pathways can be stimulated by RIG-I (207). IDO expression has been documented in other EBV-associated cancers as well (208, 209).

Tumor-associated macrophages (TAMs) infiltrate malignancies and have significant implications for the tumor microenvironment (TME) (210). TAMs may secrete IL-10 (211), an immunosuppressive agent with multiple regulatory functions, including inhibition of macrophage costimulatory molecules, Th1 cytokine production, and MHC-II antigen presentation (212). Furthermore, IL-10 secretion by TAMs is associated with worsened outcomes in a variety of malignancies (211, 213). TAM-associated IL-10 secretion is associated with EBV in GC (214), and IL-10 secretion stimulated an immunosuppressive TME with increased numbers of Tregs and impaired CD8+ T cell function. Furthermore, these patients had poorer prognoses and diminished response to fluorouracil-based chemotherapy (214). IL-10 knockdown resulted in induction of the lytic cycle and enhanced tumor cell destruction in both gastric cancer and LCLs when performed in combination with doxorubicin, suggesting that this tendency towards increased IL-10 expression can be targeted to treat EBV-associated tumors (215).

Alterations in IL-10 have also been implicated in children with EBV-associated endemic Burkitt lymphoma. Children with this disease were more likely to have monofunctional EBNA1-specific CD4+ T cells secreting IL-10. Polyfunctional CD4+ T cells secreting IFN-γ and IL-10 were less frequent. Furthermore, EBNA1-specific IFN-γ responses were only seen in 40% of patients, a much lower rate than that seen in high and low malaria, which were 84% and 66%, respectively. The authors concluded that the combination of immune regulation from IL-10 and decreased IFN-γ CD4+ T cells in EBV-associated endemic Burkitt lymphoma reduced T cell function (216).

EBV encodes a homolog of IL-10, termed viral IL-10 (vIL-10). Levels of vIL-10 and IL-6 were correlated with EBV positivity in NPC. It was shown that these two ILs caused increased expression of FOXP3 (217), a key protein in the development of Tregs (218). Consistent with these results, there was a higher proportion of CD4+CD25+ Tregs in EBV-associated NPC than healthy controls. Coculturing CD4+ T cells with the EBV-associated NPC line c666-1 resulted in a greater concentration of Tregs, increasing from 8.43% in the control group to 19.5% in the c666-1 group. Collectively, these results indicate that EBV-associated NPC can manipulate IL-6 and vIL-10 levels to augment Treg formation (217). Furthermore, vIL-10 stimulated cell cycle progression via JAK/STAT3 activation (217). It was recently demonstrated that EBV can use chemokines to attract Tregs (219). Expression of LMP1 was closely correlated with expression of the chemokines CCL17 and CCL22 (Pearson’s correlation of 0.96 and 0.95, respectively). siRNA targeting either LMP1 or LMP2A inhibited CCL17 and CCL22 production, indicating that both play a role in their induction. Supernatant obtained from Raji cells stimulated chemotaxis of Tregs. C-C chemokine receptor type 4 (CCR4) inhibition diminished this migration. Furthermore, tumors generated by injecting Raji cells into NOD-SCID mice attracted Tregs (3% of hCD45+ cells) (219). Analysis from Tumor Cancer Genome Atlas combined with published NPC RNA-Seq expression data indicated that the epithelial malignancies NPC and GC both had increased levels of CCL17, CCL22, and the Treg marker FOXP3 (219). Histopathologic examination of NPC tumors revealed a possible mixture of intrinsic and extrinsic CCL17 and CCL22 expression. Expression of LMP1 in the mouse colon cancer line CT26 resulted in an increased Treg presence, which was countered by CCR4 antagonism (219). **Table 2** summarizes mechanisms by which EBV manipulates immunoactive cellular secretions.

**Table 2:** Manipulation of immunoactive cellular secretions by EBV.

Molecule	Mechanism	Reference
IFN	Multiple	(173, 175–178, 193–197)
IL	Multiple	(198, 199)
IFN-γ	Upregulates PD-L1, IDO	(153, 158, 171)
BFRF1	Downregulate IFN-β promoter activity via inhibition of the kinase activity of IKKi	(180)
LMP2A and 2B	Increase degradation of IFN receptors	(182)
BGLF2	Inhibition of IFN-α and IFN-β signaling, including downregulation of the IFN-stimulated genes IRF1, IRF7, and ISG15	(174)
Gp110	IFN-β promoter inhibition with subsequent decreased expression of IFN signaling via inhibition of IKKi kinase polyubiquitination by TRAF3	(184)
Gp110	β-catenin degradation inhibits IFN-β production	(184)
miRNAs	Inhibit IFN and downstream signaling, for example by targeting the 3’UTR of CBP (188) or RIG-I (189)	(187–189)
RNA circBART 2.2	Decrease T cell secretion of IFN-γ via PD-L1 upregulation	(191)
BHRF1-2 miRNA	Decreased IL-1 signaling via receptor inhibition	(201)
EBER-1	Upregulate IDO production via increased RIG-I and subsequent increased TNF-α and IL-6 expression	(205, 206)
IL-6 Receptor	Upregulate IL-6 signaling to stimulate cell growth and invasion in NPC	(203)
IL-10	TAM-induced IL-10 secretion	(214)
IL-10	Inhibition of T cell function	(216)
vIL-10	Augment Treg formation	(217)
LMP1 and LMP2A	CCL17 and CCL22 secretion leads to Treg chemotaxis	(219)

### 4.6 Limiting immune recognition – MHC-I

Avoiding immune recognition is a common mechanism by which viruses avoid detection. For example, betaherpesviruses have developed numerous methods of avoiding detection by pattern recognition receptors, a key mechanism of stimulating the innate immune response (220). SARS-CoV-2 downregulates MHC-I to evade the immune system (221) and controls levels of the antigenic spike protein (222). Thus, it is unsurprising that EBV has developed mechanisms by which to minimize the ability of the host immune system to recognize infected cells.

Limiting immune recognition can be effectuated through minimizing antigen presentation, specifically through targeting MHC-I. MHC-I, also known as human leukocyte antigen (HLA)-I, complexes are key complexes of adaptive immunity (223). These molecules bind to the peptide remnants of degraded intracellular proteins and present them to CD8+ T cells. If these processed peptides are viral in origin, the cell becomes a target for the adaptive immune response (223). BILF1 is a lytic cycle gene that encodes a G-protein-coupled receptor that downregulates MHC-I expression. Indeed, BILF1-expressing B cells had 40% less MHC-I expression compared to control cells. The same set of experiments demonstrated that BILF1 expression in MJS cells resulted in a dose-dependent decrease in rates of CD8+ T cell recognition when cells were co-transfected with antigenic BZLF1 and BILF1 (224). BILF1 was associated with a decrease in the half-life of MHC-I molecules by targeting them for degradation after the MHC-I complexes populate the cell surface (224). Subsequent research demonstrated the importance of the cytoplasmic C-terminal tail of BILF1 and the intracellular tail of the HLA class I H chain in this process, the latter of which governs susceptibility to BILF1-mediated downregulation (225).

MHC class-I chain-related genes are encoded by HLA class I genes and function as ligands for natural killer group 2, member D (NKG2D) receptors seen on NK cells, γδ T cells, and CD8+ αβ T cells. Binding of the ligand and receptor lead to NK and T cell activation as well as cytokine production (226). Working with NPC cells, Wong et al. demonstrated that the EBV microRNA miR-BART7 downregulated major histocompatibility complex class I chain-related peptide A (MICA). Levels of MICA mRNA and protein were both decreased, and cells expressing miR-BART7 had lower rates of cytolysis by NK cells (227).

EBNA1 itself is an immunogenic protein that is presented to CD8+ T cells via MHC-I (228). In addition to the previously discussed downregulation of MHC-I molecules, EBV has developed stratagems to limit exposure of this protein to immune surveillance. For example, the glycine-alanine repeat domain of EBNA1 prevents mRNA translation of the EBNA1 protein, which in turn facilitates evasion of cytotoxic T cell responses (229). Nucleolin is a host cell protein implicated in this mechanism of evasion, specifically through its interaction with G-quadruplexes in the mRNA sequence encoding glycine-alanine repeat domain (230). Using a yeast model, it was further shown that C-terminal RGG motif was required for human nucleolin to bind the G-quadruplex in EBNA1 mRNA. Furthermore, type I protein arginine methyltransferases play key roles in this interaction (231).

MHC regulation is altered in EBV-associated cancers. Interestingly, MHC-I molecules were found to be upregulated in the setting of EBVaGC. In addition, genes associated with MHC-I presentation, such as TAP1, TAP2, TAPBP, and ERAP1/2, calreticulin, calnexin, and ERp57 were also increased compared to EBV-negative GC and controls. Furthermore, transcription regulators of MHC-I such as NLRC5 and RFX5 were increased. EBVaGC had higher levels of T and NK cell infiltration, which was thought to be responsible for the increased expression of MHC-I-associated genes. There was also increased IFN-γ production (232).

The above set of findings regarding increased MHC-I expression in EBVaGC has not been replicated in other EBV-associated malignancies. In fact, the opposite may be true in NPC: EBV has been associated with decreased MHC-I expression. Gene ontology analysis revealed that EBNA1 was strongly associated with decreased expression of MHC-I-associated gene classes (‘antigen presentation and endogenous antigen’, ‘MHC class I receptor activity’, and ‘antigen processing and endogenous antigen via MHC class I), with a chance probability of <10^−9^ (233). Interestingly, LMP1 has been shown to upregulate MHC-I expression in epithelial cells (234). However, it also induced c-myc, which counteracted this effect. There was no difference in expression levels of MHC-I or associated genes when comparing LMP1-positive and LMP1-negative NPC, and TAP1, tapasin, and HLA-A were all downregulated in NPC (234).

### 4.7 Limiting immune recognition – MHC-II

MHC class II molecules (MHC-II), which present exogenous proteins to CD4+ T cells (235), are also impacted by EBV. RNA-seq transcriptomic data obtained during B cell immortalization demonstrated that EBV decreased expression of several MHC-II genes. It was shown that EBNA2 was adequate to impair MHC-II transcription, and that inactivation of EBNA2 led to increases in MHC class II expression. This impaired expression led to impaired B cell mediated T cell activation. Mechanistically, it was shown that EBNA2 prevents CIITA (236), a key controller of MHC-II gene expression (237), from binding MHC-II enhancer elements. Furthermore, EBNA2 decreased CIITA transcription (236). Consistent with these results, MCH-II expression was only noted in 9/30 examined cases of EBV-positive DLBCL as compared to 49/83 in EBV-negative cases. EBV+ cases were much less likely to express CIITA and genetic changes involving CIITA were more common. EBV-positive cases also featured dysregulated BCR signaling and deficiency of antigen capture elements (238).

Like MHC-I, EBVaGC has higher levels of MHC-II expression than EBV-negative GC. EBVaGC was associated with increased mRNA levels of multiple α- and β-chains, including HLA-DPA1, -DPB1, -DQA1, -DQA2, -DQB1, -DQB2, -DRA, -DRB1, -DRB5, and -DRB6. There was increased expression of genes in the antigen presentation pathway such as cluster of differentiation (CD) 74. Furthermore, there was increased expression of transcriptional regulators such as CIITA and RFX5. This upregulation was most likely secondary to increased levels of IFNγ. Intriguingly, EBV-dependent mechanisms that inhibit IFNγ, such as LMP2A and LMP2B, did not adversely impact MHC-II upregulation (239).

**Figure 2:**
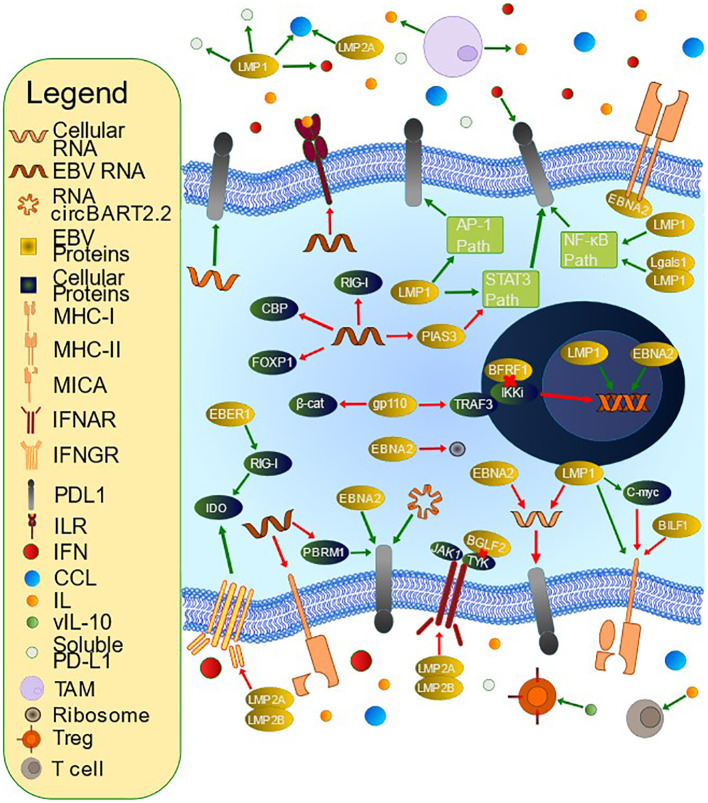
Mechanisms of T Cell Evasion by Epstein-Barr Virus (EBV). EBV has developed many mechanisms by which to evade the host immune system. PD-L1 is an immunoinhibitory molecule whose upregulation is accomplished by multiple methods. LMP1 contributes to PD-L1 upregulation via inhibition of miR-34a, interacting with Lgals1 to alter NF-kB and IRF1 signaling, inducing soluble PD-L1, upregulating IFNγ, upregulating the PD-L1 promoter and enhancer, and influencing multiple signaling pathways including JAK/STAT, AP-1, and NF-kB. EBNA2 upregulates PD-L1 through inhibition of miR-34a and promoting PD-L1 enhancer activity. miRNAs also increase PD-L1 via PBRM1 inhibition (miR-BART17-3p), targeting FOXP1 for degradation (miR-BART11), and PIAS3 downregulation leading to increased STAT3 activity (miR-BART5-5p). miR-155, a cellular miRNA, also upregulates PD-L1. EBV alters immunoactive secretions, such as IFN and IL. BFRF1 downregulates IFN-β promoter activity through IKKi inhibition, and gp110 inhibits the IFN-β promoter via inhibiting TRAF3-mediated polyubiquitination of IKKi and inhibits IFN- β via β-catenin degradation. BGLF2 inhibits both IFN-α and IFN-β, and multiple miRNAs inhibit IFN and subsequent downstream signaling, for example by targeting the 3’ untranslated region (UTR) of CBP or RIG-I. RNA circBART2.2 inhibits IFN-γ secretion via PD-L1 upregulation. EBER-1 upregulates IDO production via increased RIG-I. LMP1 and LMP2A promote CCL17 and CCL22 secretion, which stimulates Treg chemotaxis. LMP2A and LMP2B promote IFN receptor degradation. BHRF1-2 miRNA inhibits IL-1 signaling via receptor inhibition. EBV stimulates tumor-associated macrophages to secrete IL-10, which can inhibit T cells. It also encodes a viral IL-10 that can promote Treg differentiation. EBV also downregulates antigen presentation. For example, BILF1 downregulates MHC-I while miR-BART7 decreases MICA. EBNA2 inhibits MHC-II expression. LMP1-mediated upregulation of MHC-I is opposed by C-myc upregulation.

**Table 3** contains a summary of mechanisms employed by EBV to limit antigen recognition, and **Figure 2** contains a summary of mechanisms employed by EBV to evade the immune system.

**Table 3:** Mechanisms to minimize immune system recognition.

Molecule	Mechanism	Reference
BILF1	MHC-I downregulation	(224, 225)
miR-BART7	MICA downregulation	(227)
EBNA2	Inhibit MHC-II presentation by binding to prevent CIITA binding and decreases CIITA transcription	(236)
EBNA1	Contains glycine-alanine repeat domain that prevents mRNA translation of the antigenic EBNA1 protein	(229–231)
LMP1	LMP1-mediated MHC-I upregulation opposed by c-myc upregulation in epithelial cells	(234)

## 5 T cell modulation as a therapeutic option in EBV-associated malignancies

Alterations in T cells caused by EBV represent potential therapeutic targets. Perhaps the most well-researched are inhibitors of the PD-1/PD-L1 axis, which have demonstrated efficacy in a multitude of tumors (240). POLARIS-02 is a recent phase II clinical trial assessing the efficacy of the PD-1 inhibitor toripalimab in the setting of previously treated recurrent/metastatic NPC. The overall response rate (ORR) was 20.5% with an average of 12.8 months of response. Notably, a decrease in serum EBV levels by at least 50% by day 28 was associated with a much better ORR than less significant decreases of < 50% (48.3% v. 5.7%, respectively) (241). Nivolumab is a fully human PD-1 inhibitor (242) whose efficacy has also been assessed in the setting of recurrent/metastatic NPC, this time in conjunction with ipilimumab (243), a monoclonal antibody targeting the T cell inhibitor CTLA-4 (244). The best overall response rate was 38%, with a progression-free survival (PFS) of 5.3 months and an overall survival (OS) of 19.5 months. Notably, lower plasma EBV levels of <7800 IU/mL trended towards improved response rate and PFS (243).

PD-1 therapy has shown promise in EBVaGC as well. For example, 63.3% of a sample of 30 EBVaGC patients with a PD-L1 expression level of at least 1% demonstrated an overall response to PD-1 blockade, including 83.3% in patients with PD-L1 expression of at least 10% and 100% in patients with a PD-L1 expression of at least 50% (245). In addition, there is an ongoing phase II clinical trial examining the efficacy of toripalimab in addition to perioperative chemotherapy for locally advanced EBVaGC (clinical trial ID: NCT05970627).

The utility of PD-1/PD-L1 inhibition is not constrained to epithelial malignancies. *In vitro* experiments demonstrated that PD-1 expression in DLBCL inhibited T cell proliferation and impacted the T cell cytokine expression profile. PD-1 inhibition mostly reversed these adverse effects; furthermore, PD-1 inhibition was more effective in EBV+ DLBCL than EBV- DLBCL lines (246). Nivolumab has been examined as a therapeutic option for EBV-associated non-Hodgkin lymphoma (NHL) and lymphoproliferative disorders (247). ORR was 3/5 in lymphoproliferative disorder patients and 1/2 in NHL. Complete response rates (CRR) were 2/5 in the lymphoproliferative disorder and 1/2 in NHL patients (247). There is an ongoing phase II clinical trial seeking to assess the combination of the PD-1 inhibitor Sintilimab when combined with R-CHOP (rituximab, cyclophosphamide, doxorubicin, vincristine, and prednisone (248)) in previously untreated patients with EBV-associated DLBCL (clinical trial ID: NCT04181489). A separate phase II clinical trial is examining the potential role of nivolumab in EBV-associated lymphoproliferative disorders and EBV-associated NHL (clinical trial ID: NCT03258567).

The EBV proteins that modulate the immune system are being examined as potential therapeutic targets for the treatment of EBV-associated cancers (249). For example, Sinha et al. created an EBV-specific T cell expansion process that generates allogeneic T cells targeting LMP1, LMP2, and EBNA1. These cells efficiently recognized both epithelial and lymphoid malignancies associated with EBV. Xenograft mice models for NPC, gastric cancer, and lymphoma were transferred with allogeneic HLA-matched T cells specific for EBV. These mice had decreased tumor burdens and increased OS compared to mock-treated mice. Treatment responses were stronger following ‘switch therapy’ in which the third dose was changed to HLA-B7-restricted, EBNA1-specific TIG-004 T cells from HLA-A24-restricted, LMP2-specific TIG-001 T cells. Furthermore, PD-1/PD-L1 inhibition acted synergistically with the allogeneic T cells to improve treatment efficacy (250).. HLA-matched, EBV-specific T cells have also demonstrated efficacy in post-transplant lymphoproliferative disorder resistant to rituximab therapy. Patients treated with this modality experienced a complete remission or sustained partial remission in 68% of cases following hematopoietic cell transplant and in 54% of cases after solid organ transplant. One year survival was 88.9% in complete/partial response patients and 81.8% in patients with stable disease after cycle 1. 3/5 patients who experienced progressive disease after 1 cycle achieved a complete or partial remission after receiving EBV-specific T cells from a different donor (251).

As discussed above, IDO is a compound that inhibits T cell function, and it is expressed in multiple EBV-associated cancers. While there are no registered clinical trials examining its efficacy in the setting of EBV, it has been examined in other cancers. For example, a recent phase I/II clinical trial examined the efficacy of combination IDO/PD-L1 peptide vaccine with nivolumab in metastatic melanoma. The ORR was 80%, including complete responses in 43% of patients, and progression-free survival was 26 months on average. The authors did not reach median overall survival (252). In addition, there are recently completed clinical trials examining the efficacy of IDO inhibitors in solid tumors (clinical trial ID: NCT03164603 and (253)).

Vaccines that augment the T cell response to EBV antigens are also emerging as a potential therapeutic modality in EBV-associated cancers. For example, Rühl et al. examined the efficacy of heterologous prime-boost vaccination in lymphomas expressing EBV antigens (254). T cell responses were induced by generating recombinant antibodies targeting EBNA1 to dendritic via the dendritic cell receptor DEC205 (αDEC-E1). Viral vectors encoding EBNA1 or EBNA1 invariant chain, including modified vaccinia virus Ankara (MVA)-IiE1 and Adeno-E1-LMP, were also examined (254).. CD4+ and CD8+ T cell responses were elicited with heterologous vaccination. Mice were vaccinated with αDEC-E1 plus Adeno–E1-LMP or Adeno–E1-LMP plus MVA-IiE1 either prophylactically or therapeutically. In the prophylactic group, mice were challenged with EL4-E1 tumor cells (a T cell lymphoma line) two weeks after vaccination. 11/13 mice in the prophylactic group rejected the tumor cells, and survival increased from 10% to 100%. In the therapeutic group, mice were vaccinated 1-7 days after tumor cell challenge. Tumor growth was slowed, and survival increased to 75%. Interestingly, the authors found that the primary tumor site was controlled by CD4+ T cell responses, while control of distant disease sites was mediated by CD8+ T cells following heterologous prime-boost vaccination (254). Moreover, when challenged with a B cell tumor designed to mimic Burkitt lymphoma 14 days after receiving the boost, mice in the Adeno–E1-LMP plus MVA-IiE1 had lower levels of EBV DNA than PBS-treated mice, and greater than ½ the mice remained tumor free. Mice in the αDEC-E1 plus Adeno–E1-LMP group had similar EBV DNA levels to the PBS-treated control, and 35% of them remained tumor free. Following this challenge, there was no difference in the CD4+ T cell response in treated mice compared to PBS-vaccinated mice. However, Adeno–E1-LMP plus MVA-IiE1-vaccinated mice had significantly higher proportion of EBNA-1-specific CD8+ T cells. Mice vaccinated with αDEC-E1 plus Adeno–E1-LMP demonstrated only a moderate increase in CD8+ T cells (254). PD-1 levels were highest in in PBS-treated mice. PD-1 levels in αDEC-E1 plus Adeno–E1-LMP were lower than in the control, and mice vaccinated with Adeno–E1-LMP plus MVA-IiE1 had the lowest PD-1 expression level (254).

Vaccine technology is being applied in early phase clinical trials. For example, a phase I clinical trial assessed a recombinant vaccinia virus, MVA-EL, designed to augment immune responses to EBNA1 and LMP2 in the setting of NPC. Vaccination at least doubled reactivity to EBNA1 in 7 of 14 patients and to LMP2 in 6 of 14 patients, with 8 of 14 patients reacting to at least one EBV antigen by ELISpot assays (255). Using one patient who reacted to both EBNA1 and LMP2 as a sample, frequencies of EBNA1-specific CD4+ T cells and LMP2-specific CD8+ T cells were increased. Measurements of these cell populations were selected because the protein encoded by MVA-EL has EBNA1 epitopes that are predominately HLA class II-restricted and LMP2 epitopes that are predominately HLA class I-restricted. EBNA1 effectors were detected at high frequencies for over a year while LMP2-specific effectors declined. There was a greater proportion of polyfunctional T cells following vaccination (255). When combined with a similar phase IA trial done in Hong Kong, 18 of 27 patients responded to EBNA1 and 12 of 27 patients responded to LMP2, with 20 of 27 patients demonstrating an augmented T cell response to at least one antigen. All patients receiving at least dose level 3 (out of 5 total dose levels) developed a response to at least one antigen (255, 256).

There are currently clinical trials underway focusing on therapeutic vaccines, including a modified vaccinia Ankara-based vaccine targeting EBNA1 and LMP2 in patients with NPC in remission (clinical trial ID: NCT01256853). In addition, there is a phase I trial using an EBNA1 C-terminal/LMP2 chimeric protein-expressing recombinant modified vaccinia Ankara vaccine following treatment for EBV and cancer (clinical trial ID: NCT01147991). This study is seeking to establish whether vaccination induces an altered frequency of T cell responses to MHC class I and class II-restricted epitopes of LMP2 and EBNA1. It will also assess changes in plasma levels of the EBV genome. **Table 4** lists recent advances in targeting T cells in EBV-associated malignancies.

**Table 4:** Interventions targeting T Cells in EBV-associated malignancies.

Target	Intervention	Clinical Trial Status	Cancer	Reference
PD-1/PD-L1	Toripalimab	Phase II complete	NPC	(241)
PD-1/PD-L1	Nivolumab + Ipilimumab	Phase II complete	NPC	(243)
PD-1/PD-L1	PD-1 inhibitors	Literature search	EBVaGC	(245)
PD-1/PD-L1	Toripalimab + perioperative chemotherapy	Phase II ongoing	EBVaGC	NCT05970627
PD-1/PD-L1	PD-1 inhibition	Preclinical	DLBCL	(246)
PD-1/PD-L1	Nivolumab	Phase II complete	NHL/lymphoproliferative disorder	(247)
PD-1/PD-L1	Sintilimab + R-CHOP	Phase II ongoing	DLBCL	NCT04181489
PD-1/PD-L1	Nivolumab	Phase II ongoing	NHL/lymphoproliferative disorder	NCT03258567
LMP1, LMP2, EBNA1	EBV-specific T cells	Phase II complete	Multiple	(250)
EBV	EBV-specific T cells	Phase II complete	Post-transplant lymphoproliferative disorder	(251)
IDO/PD-L1*	IDO/PD-L1 peptide vaccine + nivolumab	Phase I/II complete	Metastatic melanoma	(252)
IDO*	NLG802	Phase I complete	Solid tumors	NCT03164603
IDO*	Navoximod	Phase IA complete	Solid Tumors	(253)
T cell response	Heterologous prime-boost vaccination	Preclinical	T and B lymphoma	(254)
EBNA1, LMP2	MVA	Phase I complete	NPC	(255)
EBNA1, LMP2	MVA	Phase I ongoing	NPC	NCT01256853
EBNA1 C-terminal/LMP2	MVA	Phase I ongoing	EBV and malignancy	NCT01147991

*Potential application in EBV-associated malignancies.

## 6 Concluding remarks

EBV has evolved mechanisms to evade the immune system at seemingly every turn. T cells, as primary mediators of cell mediated immunity, represent key targets of EBV’s evasion strategy. The success of this evasion strategy is manifest in the high global burden of disease. While this vast array of immunoevasive stratagems plays a critical role in viral persistence, a competent immune system is typically sufficient to prevent major complications associated with the virus. However, these strategies take on a new significance in the setting of malignancy, where they can contribute to tumor cell survival. This paper examines modalities of T cell immune evasion and discusses their implications in EBV-associated cancers.

Of note, these mechanisms may become a therapeutic target for drug development. There are already treatments targeting PD-L1, an immunosuppressive molecule upregulated by EBV, that have demonstrated efficacy in EBV-driven malignancies (78, 257). Other EBV products involved in immune evasion have emerged as potential treatments as well (258, 259). This highlights the necessity of a thorough understanding of these immunoevasive molecules, something that will continue to be important until such time as EBV-associated diseases have been fully eradicated.

## Author contributions

SD: Conceptualization, Data curation, Funding acquisition, Supervision, Writing – original draft, Writing – review & editing. PM: Data curation, Writing – original draft, Writing – review & editing. SL: Data curation, Writing – original draft, Writing – review & editing. SE: Data curation, Writing – original draft, Writing – review & editing.

## References

[1] Shannon-Lowe C Rickinson AB Bell AI. Epstein-Barr virus-associated lymphomas. Philos Trans R Soc Lond B Biol Sci (2017) 372. doi: 10.1098/rstb.2016.0271 PMC5597738 28893938

[2] Damania B Kenney SC Raab-Traub N. Epstein-Barr virus: Biology and clinical disease. Cell (2022) 185:3652–70. doi: 10.1016/j.cell.2022.08.026 PMC9529843 36113467

[3] Wong Y Meehan MT Burrows SR Doolan DL Miles JJ. Estimating the global burden of Epstein-Barr virus-related cancers. J Cancer Res Clin Oncol (2022) 148:31–46. doi: 10.1007/s00432-021-03824-y 34705104 PMC8752571

[4] Connolly SA Jardetzky TS Longnecker R. The structural basis of herpesvirus entry. Nat Rev Microbiol (2021) 19:110–21. doi: 10.1038/s41579-020-00448-w PMC8579738 33087881

[5] Gram AM Oosenbrug T Lindenbergh MF Bull C Comvalius A Dickson KJ. The Epstein-Barr Virus Glycoprotein gp150 Forms an Immune-Evasive Glycan Shield at the Surface of Infected Cells. PloS Pathog (2016) 12:e1005550. doi: 10.1371/journal.ppat.1005550 27077376 PMC4831753

[6] Odumade OA Hogquist KA Balfour HH Jr. Progress and problems in understanding and managing primary Epstein-Barr virus infections. Clin Microbiol Rev (2011) 24:193–209. doi: 10.1128/CMR.00044-10 21233512 PMC3021204

[7] Kwok H Tong AH Lin CH Lok S Farrell PJ Kwong DL. Genomic sequencing and comparative analysis of Epstein-Barr virus genome isolated from primary nasopharyngeal carcinoma biopsy. PloS One (2012) 7:e36939. doi: 10.1371/journal.pone.0036939 22590638 PMC3349645

[8] Amon W Farrell PJ. Reactivation of Epstein-Barr virus from latency. Rev Med Virol (2005) 15:149–56. doi: 10.1002/rmv.456 15546128

[9] Horwitz CA Henle W Henle G Goldfarb M Kubic P Gehrz RC. Clinical and laboratory evaluation of infants and children with Epstein-Barr virus-induced infectious mononucleosis: report of 32 patients (aged 10-48 months). Blood (1981) 57:933–8. doi: 10.1182/blood.V57.5.933.933 6260269

[10] Dunmire SK Verghese PS Balfour HH Jr. Primary Epstein-Barr virus infection. J Clin Virol (2018) 102:84–92. doi: 10.1016/j.jcv.2018.03.001 29525635

[11] Stock I. Infectious mononucleosis–a “childhood disease” of great medical concern. Med Monatsschr Pharm (2013) 36:364–8. 24266247

[12] Gomes K Goldman RD. Corticosteroids for infectious mononucleosis. Can Fam Phys (2023) 69:101–2. doi: 10.46747/cfp.6902101 PMC9945889 36813516

[13] Sausen DG Bhutta MS Gallo ES Dahari H Borenstein R. Stress-induced epstein-barr virus reactivation. Biomolecules (2021) 11. doi: 10.3390/biom11091380 PMC8470332 34572593

[14] Bjornevik K Cortese M Healy BC Kuhle J Mina MJ Leng Y. Longitudinal analysis reveals high prevalence of Epstein-Barr virus associated with multiple sclerosis. Science (2022) 375:296–301. doi: 10.1126/science.abj8222 35025605

[15] Sorgato CC Lins ESM Leao JC Vasconcelos LR Romao TP Duarte AL. EBV and CMV viral load in rheumatoid arthritis and their role in associated Sjogren’s syndrome. J Oral Pathol Med (2020) 49:693–700. doi: 10.1111/jop.13036 32428250

[16] Li ZX Zeng S Wu HX Zhou Y. The risk of systemic lupus erythematosus associated with Epstein-Barr virus infection: a systematic review and meta-analysis. Clin Exp Med (2019) 19:23–36. doi: 10.1007/s10238-018-0535-0 30361847 PMC6394567

[17] Rezk SA Zhao X Weiss LM. Epstein-Barr virus (EBV)-associated lymphoid proliferations, a 2018 update. Hum Pathol (2018) 79:18–41. doi: 10.1016/j.humpath.2018.05.020 29885408

[18] Martinez OM Krams SM. The immune response to epstein barr virus and implications for posttransplant lymphoproliferative disorder. Transplantation (2017) 101:2009–16. doi: 10.1097/TP.0000000000001767 PMC5568952 28376031

[19] Khan G Hashim MJ. Global burden of deaths from Epstein-Barr virus attributable Malignancies 1990-2010. Infect Agent Cancer (2014) 9:38. doi: 10.1186/1750-9378-9-38 25473414 PMC4253616

[20] Alosaimi MF Hoenig M Jaber F Platt CD Jones J Wallace J. Immunodeficiency and EBV-induced lymphoproliferation caused by 4-1BB deficiency. J Allergy Clin Immunol (2019) 144:574–583 e575. doi: 10.1016/j.jaci.2019.03.002 30872117 PMC6688916

[21] Long HM Meckiff BJ Taylor GS. The T-cell response to epstein-barr virus-new tricks from an old dog. Front Immunol (2019) 10:2193. doi: 10.3389/fimmu.2019.02193 31620125 PMC6759930

[22] Munz C. Immune escape by non-coding RNAs of the epstein barr virus. Front Microbiol (2021) 12:657387. doi: 10.3389/fmicb.2021.657387 34234755 PMC8257079

[23] Jiang Y Ding Y Liu S Luo B. The role of Epstein–Barr virus-encoded latent membrane proteins in host immune escape. Future Virol (2021) 16. doi: 10.2217/fvl-2020-0320

[24] Lam JKP Azzi T Hui KF Wong AMG McHugh D Caduff N. Co-infection of cytomegalovirus and epstein-barr virus diminishes the frequency of CD56(dim)NKG2A(+)KIR(-) NK cells and contributes to suboptimal control of EBV in immunosuppressed children with post-transplant lymphoproliferative disorder. Front Immunol (2020) 11:1231. doi: 10.3389/fimmu.2020.01231 32625211 PMC7311655

[25] Hammerschmidt W. The epigenetic life cycle of epstein-barr virus. Curr Top Microbiol Immunol (2015) 390:103–17. doi: 10.1007/978-3-319-22822-8_6 26424645

[26] Hatton OL Harris-Arnold A Schaffert S Krams SM Martinez OM. The interplay between Epstein-Barr virus and B lymphocytes: implications for infection, immunity, and disease. Immunol Res (2014) 58:268–76. doi: 10.1007/s12026-014-8496-1 PMC4199828 24619311

[27] Sathiyamoorthy K Jiang J Hu YX Rowe CL Mohl BS Chen J. Assembly and architecture of the EBV B cell entry triggering complex. PloS Pathog (2014) 10:e1004309. doi: 10.1371/journal.ppat.1004309 25144748 PMC4140853

[28] Chen J Longnecker R. Epithelial cell infection by Epstein-Barr virus. FEMS Microbiol Rev (2019) 43:674–83. doi: 10.1093/femsre/fuz023 PMC7317989 31584659

[29] Wei CJ Bu W Nguyen LA Batchelor JD Kim J Pittaluga S. A bivalent Epstein-Barr virus vaccine induces neutralizing antibodies that block infection and confer immunity in humanized mice. Sci Transl Med (2022) 14:eabf3685. doi: 10.1126/scitranslmed.abf3685 35507671 PMC12360862

[30] Soto AA Ortiz G Contreras S Soto-Rifo R Gonzalez PA. Role of epitranscriptomic and epigenetic modifications during the lytic and latent phases of herpesvirus infections. Microorganisms (2022) 10. doi: 10.3390/microorganisms10091754 PMC9503318 36144356

[31] Marsman C Verstegen NJ Streutker M Jorritsma T Boon L Ten Brinke A. Termination of CD40L co-stimulation promotes human B cell differentiation into antibody-secreting cells. Eur J Immunol (2022) 52:1662–75. doi: 10.1002/eji.202249972 PMC9825913 36073009

[32] Wang LW Jiang S Gewurz BE. Epstein-barr virus LMP1-mediated oncogenicity. J Virol (2017) 91. doi: 10.1128/JVI.01718-16 PMC5640852 28835489

[33] Murata T Sugimoto A Inagaki T Yanagi Y Watanabe T Sato Y. Molecular basis of epstein-barr virus latency establishment and lytic reactivation. Viruses (2021) 13. doi: 10.3390/v13122344 PMC8706188 34960613

[34] Kang MS Kieff E. Epstein-Barr virus latent genes. Exp Mol Med (2015) 47:e131. doi: 10.1038/emm.2014.84 25613728 PMC4314583

[35] Dugan JP Coleman CB Haverkos B. Opportunities to target the life cycle of epstein-barr virus (EBV) in EBV-associated lymphoproliferative disorders. Front Oncol (2019) 9:127. doi: 10.3389/fonc.2019.00127 30931253 PMC6428703

[36] Kerr JR. Epstein-Barr virus (EBV) reactivation and therapeutic inhibitors. J Clin Pathol (2019) 72:651–8. doi: 10.1136/jclinpath-2019-205822 31315893

[37] Zhang K Lv DW Li RB. Cell receptor activation and chemical induction trigger caspase-mediated cleavage of PIAS1 to facilitate epstein-barr virus reactivation. Cell Rep (2017) 21:3445–57. doi: 10.1016/j.celrep.2017.11.071 PMC5741098 29262325

[38] Rosemarie Q Sugden B. Epstein-barr virus: how its lytic phase contributes to oncogenesis. Microorganisms (2020) 8. doi: 10.3390/microorganisms8111824 PMC7699388 33228078

[39] Sugimoto A Sato Y Kanda T Murata T Narita Y Kawashima D. Different distributions of Epstein-Barr virus early and late gene transcripts within viral replication compartments. J Virol (2013) 87:6693–9. doi: 10.1128/JVI.00219-13 PMC3676136 23552415

[40] Buschle A Hammerschmidt W. Epigenetic lifestyle of Epstein-Barr virus. Semin Immunopathol (2020) 42:131–42. doi: 10.1007/s00281-020-00792-2 PMC7174264 32232535

[41] Sugden B Phelps M Domoradzki J. Epstein-Barr virus DNA is amplified in transformed lymphocytes. J Virol (1979) 31:590–5. doi: 10.1128/JVI.31.3.590-595.1979 PMC353487 229241

[42] Liang Y Zhang Y Luo B. The lytic phase of Epstein-Barr virus plays an important role in tumorigenesis. Virus Genes (2023) 59:1–12. doi: 10.1007/s11262-022-01940-6 36242711

[43] Ali A Ohashi M Casco A Djavadian R Eichelberg M Kenney SC. Rta is the principal activator of Epstein-Barr virus epithelial lytic transcription. PloS Pathog (2022) 18:e1010886. doi: 10.1371/journal.ppat.1010886 36174106 PMC9553042

[44] McKenzie J El-Guindy A. Epstein-barr virus lytic cycle reactivation. Curr Top Microbiol Immunol (2015) 391:237–61. doi: 10.1007/978-3-319-22834-1_8 26428377

[45] Weber E Buzovetsky O Heston L Yu KP Knecht KM El-Guindy A. Noncanonical basic motif of epstein-barr virus ZEBRA protein facilitates recognition of methylated DNA, high-affinity DNA binding, and lytic activation. J Virol (2019) 93. doi: 10.1128/JVI.00724-19 PMC6600195 31068430

[46] Brousset P Knecht H Rubin B Drouet E Chittal S Meggetto F. Demonstration of Epstein-Barr virus replication in Reed-Sternberg cells of Hodgkin’s disease. Blood (1993) 82:872–6. doi: 10.1182/blood.V82.3.872.872 8393354

[47] Cohen M Vistarop AG Huaman F Narbaitz M Metrebian F De Matteo E. Epstein-Barr virus lytic cycle involvement in diffuse large B cell lymphoma. Hematol Oncol (2018) 36:98–103. doi: 10.1002/hon.2465 28707331

[48] Coghill AE Proietti C Liu Z Krause L Bethony J Prokunina-Olsson L. The association between the comprehensive epstein-barr virus serologic profile and endemic burkitt lymphoma. Cancer Epidemiol Biomarkers Prev (2020) 29:57–62. doi: 10.1158/1055-9965.EPI-19-0551 31619404 PMC6954331

[49] El-Guindy A Ghiassi-Nejad M Golden S Delecluse HJ Miller G. Essential role of Rta in lytic DNA replication of Epstein-Barr virus. J Virol (2013) 87:208–23. doi: 10.1128/JVI.01995-12 PMC3536415 23077295

[50] Hammerschmidt W Sugden B. Identification and characterization of oriLyt, a lytic origin of DNA replication of Epstein-Barr virus. Cell (1988) 55:427–33. doi: 10.1016/0092-8674(88)90028-1 2846181

[51] Guo Q Qian L Guo L Shi M Chen C Lv X. Transactivators Zta and Rta of Epstein-Barr virus promote G0/G1 to S transition in Raji cells: a novel relationship between lytic virus and cell cycle. Mol Immunol (2010) 47:1783–92. doi: 10.1016/j.molimm.2010.02.017 20338640

[52] Feederle R Kost M Baumann M Janz A Drouet E Hammerschmidt W. The Epstein-Barr virus lytic program is controlled by the co-operative functions of two transactivators. EMBO J (2000) 19:3080–9. doi: 10.1093/emboj/19.12.3080 PMC203345 10856251

[53] Dremel SE Didychuk AL. Better late than never: A unique strategy for late gene transcription in the beta- and gammaherpesviruses. Semin Cell Dev Biol (2023) 146:57–69. doi: 10.1016/j.semcdb.2022.12.001 36535877 PMC10101908

[54] Gruffat H Marchione R Manet E. Herpesvirus late gene expression: A viral-specific pre-initiation complex is key. Front Microbiol (2016) 7:869. doi: 10.3389/fmicb.2016.00869 27375590 PMC4893493

[55] Chakravorty A Sugden B Johannsen EC. An epigenetic journey: epstein-barr virus transcribes chromatinized and subsequently unchromatinized templates during its lytic cycle. J Virol (2019) 93. doi: 10.1128/JVI.02247-18 PMC6450099 30700606

[56] Francis A Ragoczy T Gradoville L Heston L El-Guindy A Endo Y. Amino acid substitutions reveal distinct functions of serine 186 of the ZEBRA protein in activation of early lytic cycle genes and synergy with the Epstein-Barr virus R transactivator. J Virol (1999) 73:4543–51. doi: 10.1128/JVI.73.6.4543-4551.1999 PMC112494 10233912

[57] Hardwick JM Lieberman PM Hayward SD. A new Epstein-Barr virus transactivator, R, induces expression of a cytoplasmic early antigen. J Virol (1988) 62:2274–84. doi: 10.1128/JVI.62.7.2274-2284.1988 PMC253372 2836611

[58] Fitzsimmons L Cartlidge R Chang C Sejic N Galbraith LCA Suraweera CD. EBV BCL-2 homologue BHRF1 drives chemoresistance and lymphomagenesis by inhibiting multiple cellular pro-apoptotic proteins. Cell Death Differ (2020) 27:1554–68. doi: 10.1038/s41418-019-0435-1 PMC7206097 31645677

[59] Murayama K Nakayama S Kato-Murayama M Akasaka R Ohbayashi N Kamewari-Hayami Y. Crystal structure of epstein-barr virus DNA polymerase processivity factor BMRF1. J Biol Chem (2009) 284:35896–905. doi: 10.1074/jbc.M109.051581 PMC2791018 19801550

[60] Salamun SG Sitz J de la Cruz-Herrera CF Yockteng-Melgar J Marcon E Greenblatt J. The epstein-barr virus BMRF1 protein activates transcription and inhibits the DNA damage response by binding nuRD. J Virol (2019) 93. doi: 10.1128/JVI.01070-19 PMC6819917 31462557

[61] Bell MJ Abbott RJ Croft NP Hislop AD Burrows SR. An HLA-A2-restricted T-cell epitope mapped to the BNLF2a immune evasion protein of Epstein-Barr virus that inhibits TAP. J Virol (2009) 83:2783–8. doi: 10.1128/JVI.01724-08 PMC2648255 19129449

[62] Persson Berg L Thomsson E Hasi G Backstrom M Bergstrom T. Recombinant Epstein-Barr virus glycoprotein 350 as a serological antigen. J Virol Methods (2020) 284:113927. doi: 10.1016/j.jviromet.2020.113927 32650039

[63] Serio TR Kolman JL Miller G. Late gene expression from the Epstein-Barr virus BcLF1 and BFRF3 promoters does not require DNA replication in cis. J Virol (1997) 71:8726–34. doi: 10.1128/JVI.71.11.8726-8734.1997 PMC192337 9343231

[64] Murata T. Tegument proteins of Epstein-Barr virus: Diverse functions, complex networks, and oncogenesis. Tumour Virus Res (2023) 15:200260. doi: 10.1016/j.tvr.2023.200260 37169175 PMC10200993

[65] Yoshiyama H Shimizu N Takada K. Persistent Epstein-Barr virus infection in a human T-cell line: unique program of latent virus expression. EMBO J (1995) 14:3706–11. doi: 10.1002/j.1460-2075.1995.tb00040.x PMC394445 7641689

[66] Thorley-Lawson DA Hawkins JB Tracy SI Shapiro M. The pathogenesis of Epstein-Barr virus persistent infection. Curr Opin Virol (2013) 3:227–32. doi: 10.1016/j.coviro.2013.04.005 PMC3789532 23683686

[67] Isobe Y Sugimoto K Yang L Tamayose K Egashira M Kaneko T. Epstein-Barr virus infection of human natural killer cell lines and peripheral blood natural killer cells. Cancer Res (2004) 64:2167–74. doi: 10.1158/0008-5472.can-03-1562 15026359

[68] Phan AT Fernandez SG Somberg JJ Keck KM Miranda JL. Epstein-Barr virus latency type and spontaneous reactivation predict lytic induction levels. Biochem Biophys Res Commun (2016) 474:71–5. doi: 10.1016/j.bbrc.2016.04.070 PMC4860101 27091426

[69] Mei Y Messick TE Dheekollu J Kim HJ Molugu S Munoz LJC. Cryo-EM structure and functional studies of EBNA1 binding to the family of repeats and dyad symmetry elements of epstein-barr virus oriP. J Virol (2022) 96:e0094922. doi: 10.1128/jvi.00949-22 36037477 PMC9472633

[70] Frappier L. EBNA1 and host factors in Epstein-Barr virus latent DNA replication. Curr Opin Virol (2012) 2:733–9. doi: 10.1016/j.coviro.2012.09.005 23031715

[71] Janjetovic S Hinke J Balachandran S Akyuz N Behrmann P Bokemeyer C. Non-random pattern of integration for epstein-barr virus with preference for gene-poor genomic chromosomal regions into the genome of burkitt lymphoma cell lines. Viruses (2022) 14. doi: 10.3390/v14010086 PMC8781420 35062290

[72] Wang C Li D Zhang L Jiang S Liang J Narita Y. RNA sequencing analyses of gene expression during epstein-barr virus infection of primary B lymphocytes. J Virol (2019) 93. doi: 10.1128/JVI.00226-19 PMC6580941 31019051

[73] Price AM Luftig MA. To be or not IIb: a multi-step process for Epstein-Barr virus latency establishment and consequences for B cell tumorigenesis. PloS Pathog (2015) 11:e1004656. doi: 10.1371/journal.ppat.1004656 25790223 PMC4366242

[74] Wang F Tsang SF Kurilla MG Cohen JI Kieff E. Epstein-Barr virus nuclear antigen 2 transactivates latent membrane protein LMP1. J Virol (1990) 64:3407–16. doi: 10.1128/JVI.64.7.3407-3416.1990 PMC249594 2352328

[75] Wang L Grossman SR Kieff E. Epstein-Barr virus nuclear protein 2 interacts with p300, CBP, and PCAF histone acetyltransferases in activation of the LMP1 promoter. Proc Natl Acad Sci U.S.A. (2000) 97:430–5. doi: 10.1073/pnas.97.1.430 PMC26680 10618435

[76] Price AM Luftig MA. Dynamic Epstein-Barr virus gene expression on the path to B-cell transformation. Adv Virus Res (2014) 88:279–313. doi: 10.1016/B978-0-12-800098-4.00006-4 24373315 PMC4911173

[77] Murata T Sato Y Kimura H. Modes of infection and oncogenesis by the Epstein-Barr virus. Rev Med Virol (2014) 24:242–53. doi: 10.1002/rmv.1786 24578255

[78] Sausen DG Basith A Muqeemuddin S. EBV and lymphomagenesis. Cancers (Basel) (2023) 15. doi: 10.3390/cancers15072133 PMC10093459 37046794

[79] Hodin TL Najrana T Yates JL. Efficient replication of Epstein-Barr virus-derived plasmids requires tethering by EBNA1 to host chromosomes. J Virol (2013) 87:13020–8. doi: 10.1128/JVI.01606-13 PMC3838108 24067969

[80] Sears J Ujihara M Wong S Ott C Middeldorp J Aiyar A. The amino terminus of Epstein-Barr Virus (EBV) nuclear antigen 1 contains AT hooks that facilitate the replication and partitioning of latent EBV genomes by tethering them to cellular chromosomes. J Virol (2004) 78:11487–505. doi: 10.1128/JVI.78.21.11487-11505.2004 PMC523237 15479791

[81] Frappier L. Ebna1. Curr Top Microbiol Immunol (2015) 391:3–34. doi: 10.1007/978-3-319-22834-1_1 26428370

[82] Westhoff Smith D Chakravorty A Hayes M Hammerschmidt W Sugden B. The epstein-barr virus oncogene EBNA1 suppresses natural killer cell responses and apoptosis early after infection of peripheral B cells. mBio (2021) 12:e0224321. doi: 10.1128/mBio.02243-21 34781735 PMC8593684

[83] Blake N Lee S Redchenko I Thomas W Steven N Leese A. Human CD8+ T cell responses to EBV EBNA1: HLA class I presentation of the (Gly-Ala)-containing protein requires exogenous processing. Immunity (1997) 7:791–802. doi: 10.1016/s1074-7613(00)80397-0 9430224

[84] Alfieri C Birkenbach M Kieff E. Early events in Epstein-Barr virus infection of human B lymphocytes. Virology (1991) 181:595–608. doi: 10.1016/0042-6822(91)90893-g 1849678

[85] Pich D Mrozek-Gorska P Bouvet M Sugimoto A Akidil E Grundhoff A. First days in the life of naive human B lymphocytes infected with epstein-barr virus. mBio (2019) 10. doi: 10.1128/mBio.01723-19 PMC6751056 31530670

[86] Kempkes B Ling PD. EBNA2 and Its Coactivator EBNA-LP. In: Münz C, editor. Epstein Barr Virus Volume 2. Current Topics in Microbiology and Immunology, vol. 391 . Cham: Springer (2015). p. 35–59. 10.1007/978-3-319-22834-1_2 26428371

[87] Saha A Robertson ES. Mechanisms of B-cell oncogenesis induced by epstein-barr virus. J Virol (2019) 93. doi: 10.1128/JVI.00238-19 PMC6580952 30971472

[88] Beer S Wange LE Zhang X Kuklik-Roos C Enard W Hammerschmidt W. EBNA2-EBF1 complexes promote MYC expression and metabolic processes driving S-phase progression of Epstein-Barr virus-infected B cells. Proc Natl Acad Sci U.S.A. (2022) 119:e2200512119. doi: 10.1073/pnas.2200512119 35857872 PMC9335265

[89] Pages F Galon J Karaschuk G Dudziak D Camus M Lazar V. Epstein-Barr virus nuclear antigen 2 induces interleukin-18 receptor expression in B cells. Blood (2005) 105:1632–9. doi: 10.1182/blood-2004-08-3196 15498855

[90] Nold-Petry CA Lo CY Rudloff I Elgass KD Li S Gantier MP. IL-37 requires the receptors IL-18Ralpha and IL-1R8 (SIGIRR) to carry out its multifaceted anti-inflammatory program upon innate signal transduction. Nat Immunol (2015) 16:354–65. doi: 10.1038/ni.3103 25729923

[91] Bhattacharjee S Ghosh Roy S Bose P Saha A. Role of EBNA-3 family proteins in EBV associated B-cell lymphomagenesis. Front Microbiol (2016) 7:457. doi: 10.3389/fmicb.2016.00457 27092119 PMC4824013

[92] Tomkinson B Robertson E Kieff E. Epstein-Barr virus nuclear proteins EBNA-3A and EBNA-3C are essential for B-lymphocyte growth transformation. J Virol (1993) 67:2014–25. doi: 10.1128/JVI.67.4.2014-2025.1993 PMC240270 8445720

[93] Chen A Divisconte M Jiang X Quink C Wang F. Epstein-Barr virus with the latent infection nuclear antigen 3B completely deleted is still competent for B-cell growth transformation *in vitro* . J Virol (2005) 79:4506–9. doi: 10.1128/JVI.79.7.4506-4509.2005 PMC1061580 15767450

[94] Styles CT Paschos K White RE Farrell PJ. The cooperative functions of the EBNA3 proteins are central to EBV persistence and latency. Pathogens (2018) 7. doi: 10.3390/pathogens7010031 PMC5874757 29562595

[95] Szymula A Palermo RD Bayoumy A Groves IJ Ba Abdullah M Holder B. Epstein-Barr virus nuclear antigen EBNA-LP is essential for transforming naive B cells, and facilitates recruitment of transcription factors to the viral genome. PloS Pathog (2018) 14:e1006890. doi: 10.1371/journal.ppat.1006890 29462212 PMC5834210

[96] Kilger E Kieser A Baumann M Hammerschmidt W. Epstein-Barr virus-mediated B-cell proliferation is dependent upon latent membrane protein 1, which simulates an activated CD40 receptor. EMBO J (1998) 17:1700–9. doi: 10.1093/emboj/17.6.1700 PMC1170517 9501091

[97] Laman JD Claassen E Noelle RJ. Functions of CD40 and its ligand, gp39 (CD40L). Crit Rev Immunol (2017) 37:371–420. doi: 10.1615/CritRevImmunol.v37.i2-6.100 29773027

[98] Wang L Ning S. New look of EBV LMP1 signaling landscape. Cancers (Basel) (2021) 13. doi: 10.3390/cancers13215451 PMC8582580 34771613

[99] Lavorgna A Harhaj EW. EBV LMP1: New and shared pathways to NF-kappaB activation. Proc Natl Acad Sci U.S.A. (2012) 109:2188–9. doi: 10.1073/pnas.1121357109 PMC3289357 22308477

[100] Kung CP Meckes DG Jr. Raab-Traub N. Epstein-Barr virus LMP1 activates EGFR, STAT3, and ERK through effects on PKCdelta. J Virol (2011) 85:4399–408. doi: 10.1128/JVI.01703-10 PMC3126279 21307189

[101] Tsai CL Li HP Lu YJ Hsueh C Liang Y Chen CL. Activation of DNA methyltransferase 1 by EBV LMP1 Involves c-Jun NH(2)-terminal kinase signaling. Cancer Res (2006) 66:11668–76. doi: 10.1158/0008-5472.CAN-06-2194 17178861

[102] Kume A Shinozaki-Ushiku A Kunita A Kondo A Ushiku T. Enhanced PD-L1 expression in LMP1-positive cells of epstein-barr virus-associated malignant lymphomas and lymphoproliferative disorders: A single-cell resolution analysis with multiplex fluorescence immunohistochemistry and *in situ* hybridization. Am J Surg Pathol (2022) 46:1386–96. doi: 10.1097/PAS.0000000000001919 35605962

[103] Tsai CY Sakakibara S Yasui T Minamitani T Okuzaki D Kikutani H. Bystander inhibition of humoral immune responses by Epstein-Barr virus LMP1. Int Immunol (2018) 30:579–90. doi: 10.1093/intimm/dxy053 30137504

[104] Chen ST Oliveira TY Gazumyan A Cipolla M Nussenzweig MC. B cell receptor signaling in germinal centers prolongs survival and primes B cells for selection. Immunity (2023) 56:547–561 e547. doi: 10.1016/j.immuni.2023.02.003 36882061 PMC10424567

[105] Fuentes-Panana EM Bannish G Monroe JG. Basal B-cell receptor signaling in B lymphocytes: mechanisms of regulation and role in positive selection, differentiation, and peripheral survival. Immunol Rev (2004) 197:26–40. doi: 10.1111/j.0105-2896.2004.0105.x 14962184

[106] Mancao C Hammerschmidt W. Epstein-Barr virus latent membrane protein 2A is a B-cell receptor mimic and essential for B-cell survival. Blood (2007) 110:3715–21. doi: 10.1182/blood-2007-05-090142 PMC2077319 17682125

[107] Fish K Comoglio F Shaffer AL 3rd Ji Y Pan KT Scheich S. Rewiring of B cell receptor signaling by Epstein-Barr virus LMP2A. Proc Natl Acad Sci U.S.A. (2020) 117:26318–27. doi: 10.1073/pnas.2007946117 PMC7584892 33020271

[108] Lin JH Lin JY Chou YC Chen MR Yeh TH Lin CW. Epstein-Barr virus LMP2A suppresses MHC class II expression by regulating the B-cell transcription factors E47 and PU.1. Blood (2015) 125:2228–38. doi: 10.1182/blood-2014-08-594689 25631773

[109] Fruehling S Longnecker R. The immunoreceptor tyrosine-based activation motif of Epstein-Barr virus LMP2A is essential for blocking BCR-mediated signal transduction. Virology (1997) 235:241–51. doi: 10.1006/viro.1997.8690 9281504

[110] Rovedo M Longnecker R. Epstein-barr virus latent membrane protein 2B (LMP2B) modulates LMP2A activity. J Virol (2007) 81:84–94. doi: 10.1128/JVI.01302-06 17035319 PMC1797235

[111] Rechsteiner MP Berger C Zauner L Sigrist JA Weber M Longnecker R. Latent membrane protein 2B regulates susceptibility to induction of lytic Epstein-Barr virus infection. J Virol (2008) 82:1739–47. doi: 10.1128/JVI.01723-07 PMC2258708 18057232

[112] Marshall JS Warrington R Watson W Kim HL. An introduction to immunology and immunopathology. Allergy Asthma Clin Immunol (2018) 14:49. doi: 10.1186/s13223-018-0278-1 30263032 PMC6156898

[113] Kloetzel PM. The proteasome and MHC class I antigen processing. Biochim Biophys Acta (2004) 1695:225–33. doi: 10.1016/j.bbamcr.2004.10.004 15571818

[114] Roche PA Furuta K. The ins and outs of MHC class II-mediated antigen processing and presentation. Nat Rev Immunol (2015) 15:203–16. doi: 10.1038/nri3818 PMC6314495 25720354

[115] Rock KL Reits E Neefjes J. Present yourself! By MHC class I and MHC class II molecules. Trends Immunol (2016) 37:724–37. doi: 10.1016/j.it.2016.08.010 PMC5159193 27614798

[116] Laidlaw BJ Craft JE Kaech SM. The multifaceted role of CD4(+) T cells in CD8(+) T cell memory. Nat Rev Immunol (2016) 16:102–11. doi: 10.1038/nri.2015.10 PMC4860014 26781939

[117] Luckheeram RV Zhou R Verma AD Xia B. CD4(+)T cells: differentiation and functions. Clin Dev Immunol (2012) 2012:925135. doi: 10.1155/2012/925135 22474485 PMC3312336

[118] Dowell AC Haigh TA Ryan GB Turner JE Long HM Taylor GS. Cytotoxic CD4+ T-cells specific for EBV capsid antigen BORF1 are maintained in long-term latently infected healthy donors. PloS Pathog (2021) 17:e1010137. doi: 10.1371/journal.ppat.1010137 34882759 PMC8691624

[119] Meckiff BJ Ladell K McLaren JE Ryan GB Leese AM James EA. Primary EBV infection induces an acute wave of activated antigen-specific cytotoxic CD4(+) T cells. J Immunol (2019) 203:1276–87. doi: 10.4049/jimmunol.1900377 PMC6697742 31308093

[120] Olotu FA Soliman MES. Immunoinformatics prediction of potential B-cell and T-cell epitopes as effective vaccine candidates for eliciting immunogenic responses against Epstein-Barr virus. BioMed J (2021) 44:317–37. doi: 10.1016/j.bj.2020.01.002 PMC8358216 34154948

[121] Schneider-Hohendorf T Gerdes LA Pignolet B Gittelman R Ostkamp P Rubelt F. Broader Epstein-Barr virus-specific T cell receptor repertoire in patients with multiple sclerosis. J Exp Med (2022) 219. doi: 10.1084/jem.20220650 PMC9437111 36048016

[122] Lanz TV Brewer RC Ho PP Moon JS Jude KM Fernandez D. Clonally expanded B cells in multiple sclerosis bind EBV EBNA1 and GlialCAM. Nature (2022) 603:321–7. doi: 10.1038/s41586-022-04432-7 PMC9382663 35073561

[123] Hislop AD Taylor GS. T-cell responses to EBV. Curr Top Microbiol Immunol (2015) 391:325–53. doi: 10.1007/978-3-319-22834-1_11 26428380

[124] Deng Y Chatterjee B Zens K Zdimerova H Muller A Schuhmachers P. CD27 is required for protective lytic EBV antigen-specific CD8+ T-cell expansion. Blood (2021) 137:3225–36. doi: 10.1182/blood.2020009482 PMC8351885 33827115

[125] Chijioke O Azzi T Nadal D Munz C. Innate immune responses against Epstein Barr virus infection. J Leukoc Biol (2013) 94:1185–90. doi: 10.1189/jlb.0313173 PMC3828602 23812328

[126] Hollsberg P Hansen HJ Haahr S. Altered CD8+ T cell responses to selected Epstein-Barr virus immunodominant epitopes in patients with multiple sclerosis. Clin Exp Immunol (2003) 132:137–43. doi: 10.1046/j.1365-2249.2003.02114.x PMC1808679 12653848

[127] Lam JKP Hui KF Ning RJ Xu XQ Chan KH Chiang AKS. Emergence of CD4+ and CD8+ Polyfunctional T cell responses against immunodominant lytic and latent EBV antigens in children with primary EBV infection. Front Microbiol (2018) 9:416. doi: 10.3389/fmicb.2018.00416 29599759 PMC5863510

[128] Pudney VA Leese AM Rickinson AB Hislop AD. CD8+ immunodominance among Epstein-Barr virus lytic cycle antigens directly reflects the efficiency of antigen presentation in lytically infected cells. J Exp Med (2005) 201:349–60. doi: 10.1084/jem.20041542 PMC2213038 15684323

[129] Hislop AD Ressing ME van Leeuwen D Pudney VA Horst D Koppers-Lalic D. CD8+ T cell immune evasion protein specific to Epstein-Barr virus and its close relatives in Old World primates. J Exp Med (2007) 204:1863–73. doi: 10.1084/jem.20070256 PMC2118677 17620360

[130] Woodberry T Suscovich TJ Henry LM Davis JK Frahm N Walker BD. Differential targeting and shifts in the immunodominance of Epstein-Barr virus–specific CD8 and CD4 T cell responses during acute and persistent infection. J Infect Dis (2005) 192:1513–24. doi: 10.1086/491741 16206065

[131] Rickinson AB Moss DJ. Human cytotoxic T lymphocyte responses to Epstein-Barr virus infection. Annu Rev Immunol (1997) 15:405–31. doi: 10.1146/annurev.immunol.15.1.405 9143694

[132] Long HM Leese AM Chagoury OL Connerty SR Quarcoopome J Quinn LL. Cytotoxic CD4+ T cell responses to EBV contrast with CD8 responses in breadth of lytic cycle antigen choice and in lytic cycle recognition. J Immunol (2011) 187:92–101. doi: 10.4049/jimmunol.1100590 21622860 PMC3154640

[133] Adhikary D Behrends U Boerschmann H Pfunder A Burdach S Moosmann A. Immunodominance of lytic cycle antigens in Epstein-Barr virus-specific CD4+ T cell preparations for therapy. PloS One (2007) 2:e583. doi: 10.1371/journal.pone.0000583 17611619 PMC1894652

[134] Munz C Bickham KL Subklewe M Tsang ML Chahroudi A Kurilla MG. Human CD4(+) T lymphocytes consistently respond to the latent Epstein-Barr virus nuclear antigen EBNA1. J Exp Med (2000) 191:1649–60. doi: 10.1084/jem.191.10.1649 PMC2193162 10811859

[135] Leung CS Haigh TA Mackay LK Rickinson AB Taylor GS. Nuclear location of an endogenously expressed antigen, EBNA1, restricts access to macroautophagy and the range of CD4 epitope display. Proc Natl Acad Sci U.S.A. (2010) 107:2165–70. doi: 10.1073/pnas.0909448107 PMC2836662 20133861

[136] Leen A Meij P Redchenko I Middeldorp J Bloemena E Rickinson A. Differential immunogenicity of Epstein-Barr virus latent-cycle proteins for human CD4(+) T-helper 1 responses. J Virol (2001) 75:8649–59. doi: 10.1128/jvi.75.18.8649-8659.2001 PMC115110 11507210

[137] Shechter O Sausen DG Gallo ES Dahari H Borenstein R. Epstein-barr virus (EBV) epithelial associated Malignancies: exploring pathologies and current treatments. Int J Mol Sci (2022) 23. doi: 10.3390/ijms232214389 PMC9699474 36430864

[138] Bauer M Jasinski-Bergner S Mandelboim O Wickenhauser C Seliger B. Epstein-barr virus-associated Malignancies and immune escape: the role of the tumor microenvironment and tumor cell evasion strategies. Cancers (Basel) (2021) 13. doi: 10.3390/cancers13205189 PMC8533749 34680337

[139] Hudson K Cross N Jordan-Mahy N Leyland R. The extrinsic and intrinsic roles of PD-L1 and its receptor PD-1: implications for immunotherapy treatment. Front Immunol (2020) 11:568931. doi: 10.3389/fimmu.2020.568931 33193345 PMC7609400

[140] Fanelli G Romano M Nova-Lamperti E Werner Sunderland M Nerviani A Scotta C. PD-L1 signaling on human memory CD4+ T cells induces a regulatory phenotype. PloS Biol (2021) 19:e3001199. doi: 10.1371/journal.pbio.3001199 33901179 PMC8101994

[141] Green MR Rodig S Juszczynski P Ouyang J Sinha P O’Donnell E. Constitutive AP-1 activity and EBV infection induce PD-L1 in Hodgkin lymphomas and posttransplant lymphoproliferative disorders: implications for targeted therapy. Clin Cancer Res (2012) 18:1611–8. doi: 10.1158/1078-0432.CCR-11-1942 PMC3321508 22271878

[142] Yanagi Y Okuno Y Narita Y Masud H Watanabe T Sato Y. RNAseq analysis identifies involvement of EBNA2 in PD-L1 induction during Epstein-Barr virus infection of primary B cells. Virology (2021) 557:44–54. doi: 10.1016/j.virol.2021.02.004 33639481

[143] Anastasiadou E Stroopinsky D Alimperti S Jiao AL Pyzer AR Cippitelli C. Epstein-Barr virus-encoded EBNA2 alters immune checkpoint PD-L1 expression by downregulating miR-34a in B-cell lymphomas. Leukemia (2019) 33:132–47. doi: 10.1038/s41375-018-0178-x PMC6327052 29946193

[144] Yanagi Y Hara Y Mabuchi S Watanabe T Sato Y Kimura H. PD-L1 upregulation by lytic induction of Epstein-Barr Virus. Virology (2022) 568:31–40. doi: 10.1016/j.virol.2022.01.006 35093708

[145] Cha JH Chan LC Li CW Hsu JL Hung MC. Mechanisms controlling PD-L1 expression in cancer. Mol Cell (2019) 76:359–70. doi: 10.1016/j.molcel.2019.09.030 PMC6981282 31668929

[146] Lin Q Wang X Hu Y. The opportunities and challenges in immunotherapy: Insights from the regulation of PD-L1 in cancer cells. Cancer Lett (2023) 569:216318. doi: 10.1016/j.canlet.2023.216318 37454966

[147] Han Y Liu D Li L. PD-1/PD-L1 pathway: current researches in cancer. Am J Cancer Res (2020) 10:727–42. PMC7136921 32266087

[148] Dias JM Santana IVV da Silva VD Carvalho AL Arantes L. Analysis of epstein-barr virus (EBV) and PD-L1 expression in nasopharyngeal carcinoma patients in a non-endemic region. Int J Mol Sci (2022) 23. doi: 10.3390/ijms231911720 PMC9569432 36233023

[149] Qian X Chen H Tao Y. Biomarkers predicting clinical outcomes in nasopharyngeal cancer patients receiving immune checkpoint inhibitors: A systematic review and meta-analysis. Front Immunol (2023) 14:1146898. doi: 10.3389/fimmu.2023.1146898 37063822 PMC10102485

[150] Xu JY Wei XL Ren C Zhang Y Hu YF Li JY. Association of plasma epstein-barr virus DNA with outcomes for patients with recurrent or metastatic nasopharyngeal carcinoma receiving anti-programmed cell death 1 immunotherapy. JAMA Netw Open (2022) 5:e220587. doi: 10.1001/jamanetworkopen.2022.0587 35230439 PMC8889459

[151] Sun Q Fu Y Chen X Li L Wu H Liu Y. Prognostic perspectives of STING and PD-L1 expression and correlation with the prognosis of epstein-barr virus-associated gastric cancers. Gut Liver (2022) 16:875–91. doi: 10.5009/gnl210359 PMC9668503 35611665

[152] Lima A Sousa H Medeiros R Nobre A MaChado M. PD-L1 expression in EBV associated gastric cancer: a systematic review and meta-analysis. Discovery Oncol (2022) 13:19. doi: 10.1007/s12672-022-00479-0 PMC8941030 35318527

[153] Fang W Zhang J Hong S Zhan J Chen N Qin T. EBV-driven LMP1 and IFN-gamma up-regulate PD-L1 in nasopharyngeal carcinoma: Implications for oncotargeted therapy. Oncotarget (2014) 5:12189–202. doi: 10.18632/oncotarget.2608 PMC4322961 25361008

[154] Yixing C Xie F Chen J Dayu L Li Z Luo Q. The LMP1/Lgals1-NF-Kb-IRF1-PDL1 axis promotes immune escape in nasopharyngeal carcinoma, IJROBP (2022) 114(3):e514. doi: 10.1016/j.ijrobp.2022.07.2092

[155] Niu M Liu Y Yi M Jiao D Wu K. Biological characteristics and clinical significance of soluble PD-1/PD-L1 and exosomal PD-L1 in cancer. Front Immunol (2022) 13:827921. doi: 10.3389/fimmu.2022.827921 35386715 PMC8977417

[156] Bailly C Thuru X Quesnel B. Soluble programmed death ligand-1 (sPD-L1): A pool of circulating proteins implicated in health and diseases. Cancers (Basel) (2021) 13. doi: 10.3390/cancers13123034 PMC8233757 34204509

[157] Kase K Kondo S Wakisaka N Dochi H Mizokami H Kobayashi E. Epstein-barr virus LMP1 induces soluble PD-L1 in nasopharyngeal carcinoma. Microorganisms (2021) 9. doi: 10.3390/microorganisms9030603 PMC7998736 33804064

[158] Sasaki S Nishikawa J Sakai K Iizasa H Yoshiyama H Yanagihara M. EBV-associated gastric cancer evades T-cell immunity by PD-1/PD-L1 interactions. Gastric Cancer (2019) 22:486–96. doi: 10.1007/s10120-018-0880-4 30264329

[159] Yoon CJ Chang MS Kim DH Kim W Koo BK Yun SC. Epstein-Barr virus-encoded miR-BART5-5p upregulates PD-L1 through PIAS3/pSTAT3 modulation, worsening clinical outcomes of PD-L1-positive gastric carcinomas. Gastric Cancer (2020) 23:780–95. doi: 10.1007/s10120-020-01059-3 32206940

[160] Nakayama A Abe H Kunita A Saito R Kanda T Yamashita H. Viral loads correlate with upregulation of PD-L1 and worse patient prognosis in Epstein-Barr Virus-associated gastric carcinoma. PloS One (2019) 14:e0211358. doi: 10.1371/journal.pone.0211358 30695048 PMC6350976

[161] Wang J Ge J Wang Y Xiong F Guo J Jiang X. EBV miRNAs BART11 and BART17-3p promote immune escape through the enhancer-mediated transcription of PD-L1. Nat Commun (2022) 13:866. doi: 10.1038/s41467-022-28479-2 35165282 PMC8844414

[162] Satou A Nakamura S. EBV-positive B-cell lymphomas and lymphoproliferative disorders: Review from the perspective of immune escape and immunodeficiency. Cancer Med (2021) 10:6777–85. doi: 10.1002/cam4.4198 PMC8495296 34387382

[163] Paydas S Bagir E Seydaoglu G Ercolak V Ergin M. Programmed death-1 (PD-1), programmed death-ligand 1 (PD-L1), and EBV-encoded RNA (EBER) expression in Hodgkin lymphoma. Ann Hematol (2015) 94:1545–52. doi: 10.1007/s00277-015-2403-2 26004934

[164] Bi XW Wang H Zhang WW Wang JH Liu WJ Xia ZJ. PD-L1 is upregulated by EBV-driven LMP1 through NF-kappaB pathway and correlates with poor prognosis in natural killer/T-cell lymphoma. J Hematol Oncol (2016) 9:109. doi: 10.1186/s13045-016-0341-7 27737703 PMC5064887

[165] Qiu L Zheng H Zhao X. The prognostic and clinicopathological significance of PD-L1 expression in patients with diffuse large B-cell lymphoma: a meta-analysis. BMC Cancer (2019) 19:273. doi: 10.1186/s12885-019-5466-y 30917792 PMC6437873

[166] Frontzek F Staiger AM Wullenkord R Grau M Zapukhlyak M Kurz KS. Molecular profiling of EBV associated diffuse large B-cell lymphoma. Leukemia (2023) 37:670–9. doi: 10.1038/s41375-022-01804-w PMC9991915 36604606

[167] Panjwani PK Charu V DeLisser M Molina-Kirsch H Natkunam Y Zhao S. Programmed death-1 ligands PD-L1 and PD-L2 show distinctive and restricted patterns of expression in lymphoma subtypes. Hum Pathol (2018) 71:91–9. doi: 10.1016/j.humpath.2017.10.029 29122656

[168] Takahara T Satou A Ishikawa E Kohno K Kato S Suzuki Y. Clinicopathological analysis of neoplastic PD-L1-positive EBV(+) diffuse large B cell lymphoma, not otherwise specified, in a Japanese cohort. Virchows Arch (2021) 478:541–52. doi: 10.1007/s00428-020-02901-w 32803453

[169] Mahesh G Biswas R. MicroRNA-155: A master regulator of inflammation. J Interferon Cytokine Res (2019) 39:321–30. doi: 10.1089/jir.2018.0155 PMC6591773 30998423

[170] Zheng Z Sun R Zhao HJ Fu D Zhong HJ Weng XQ. MiR155 sensitized B-lymphoma cells to anti-PD-L1 antibody via PD-1/PD-L1-mediated lymphoma cell interaction with CD8+T cells. Mol Cancer (2019) 18:54. doi: 10.1186/s12943-019-0977-3 30925928 PMC6441197

[171] Chakravorty S Yan B Wang C Wang L Quaid JT Lin CF. Integrated pan-cancer map of EBV-associated neoplasms reveals functional host-virus interactions. Cancer Res (2019) 79:6010–23. doi: 10.1158/0008-5472.CAN-19-0615 PMC6891172 31481499

[172] Cone AS York SB Meckes DG Jr. Extracellular vesicles in epstein-barr virus pathogenesis. Curr Clin Microbiol Rep (2019) 6:121–31. doi: 10.1007/s40588-019-00123-6 PMC7015464 32051811

[173] Zhou H Jing S Liu Y Wang X Duan X Xiong W. Identifying the key genes of Epstein-Barr virus-regulated tumour immune microenvironment of gastric carcinomas. Cell Prolif (2023) 56:e13373. doi: 10.1111/cpr.13373 36519208 PMC9977676

[174] Liu X Sadaoka T Krogmann T Cohen JI. Epstein-barr virus (EBV) tegument protein BGLF2 suppresses type I interferon signaling to promote EBV reactivation. J Virol (2020) 94. doi: 10.1128/JVI.00258-20 PMC7269453 32213613

[175] Crouse J Kalinke U Oxenius A. Regulation of antiviral T cell responses by type I interferons. Nat Rev Immunol (2015) 15:231–42. doi: 10.1038/nri3806 25790790

[176] Kuka M De Giovanni M Iannacone M. The role of type I interferons in CD4(+) T cell differentiation. Immunol Lett (2019) 215:19–23. doi: 10.1016/j.imlet.2019.01.013 30771379 PMC7234836

[177] Eskandari SK Allos H Safadi JM Sulkaj I Sanders JSF P. C. Type I interferons augment regulatory T cell polarization in concert with ancillary cytokine signals. Front Transplant (2023) 2:1149334. doi: 10.3389/frtra.2023.1149334 PMC11235373 38993887

[178] Kolumam GA Thomas S Thompson LJ Sprent J Murali-Krishna K. Type I interferons act directly on CD8 T cells to allow clonal expansion and memory formation in response to viral infection. J Exp Med (2005) 202:637–50. doi: 10.1084/jem.20050821 PMC2212878 16129706

[179] Rex V Zargari R Stempel M Halle S Brinkmann MM. The innate and T-cell mediated immune response during acute and chronic gammaherpesvirus infection. Front Cell Infect Microbiol (2023) 13:1146381. doi: 10.3389/fcimb.2023.1146381 37065193 PMC10102517

[180] Wang P Deng Y Guo Y Xu Z Li Y Ou X. Epstein-barr virus early protein BFRF1 suppresses IFN-beta activity by inhibiting the activation of IRF3. Front Immunol (2020) 11:513383. doi: 10.3389/fimmu.2020.513383 33391252 PMC7774019

[181] Wang JT Doong SL Teng SC Lee CP Tsai CH Chen MR. Epstein-Barr virus BGLF4 kinase suppresses the interferon regulatory factor 3 signaling pathway. J Virol (2009) 83:1856–69. doi: 10.1128/JVI.01099-08 PMC2643756 19052084

[182] Shah KM Stewart SE Wei W Woodman CB O’Neil JD Dawson CW. The EBV-encoded latent membrane proteins, LMP2A and LMP2B, limit the actions of interferon by targeting interferon receptors for degradation. Oncogene (2009) 28:3903–14. doi: 10.1038/onc.2009.249 PMC2774296 19718044

[183] Neuhierl B Feederle R Hammerschmidt W Delecluse HJ. Glycoprotein gp110 of Epstein-Barr virus determines viral tropism and efficiency of infection. Proc Natl Acad Sci U.S.A. (2002) 99:15036–41. doi: 10.1073/pnas.232381299 PMC137540 12409611

[184] Guo Y Pan L Wang L Wang S Fu J Luo W. Epstein-Barr Virus Envelope Glycoprotein gp110 Inhibits IKKi-Mediated Activation of NF-kappaB and Promotes the Degradation of beta-Catenin. Microbiol Spectr (2023) 11:e0032623. doi: 10.1128/spectrum.00326-23 37022262 PMC10269791

[185] Hillesheim A Nordhoff C Boergeling Y Ludwig S Wixler V. beta-catenin promotes the type I IFN synthesis and the IFN-dependent signaling response but is suppressed by influenza A virus-induced RIG-I/NF-kappaB signaling. Cell Commun Signal (2014) 12:29. doi: 10.1186/1478-811X-12-29 24767605 PMC4021428

[186] Marineau A Khan KA Servant MJ. Roles of GSK-3 and beta-catenin in antiviral innate immune sensing of nucleic acids. Cells (2020) 9. doi: 10.3390/cells9040897 PMC7226782 32272583

[187] Bouvet M Voigt S Tagawa T Albanese M Chen YA Chen Y. Multiple Viral microRNAs Regulate Interferon Release and Signaling Early during Infection with Epstein-Barr Virus. mBio (2021) 12. doi: 10.1128/mBio.03440-20 PMC8092300 33785626

[188] Hooykaas MJG van Gent M Soppe JA Kruse E Boer IGJ van Leenen D. EBV microRNA BART16 suppresses type I IFN signaling. J Immunol (2017) 198:4062–73. doi: 10.4049/jimmunol.1501605 28416598

[189] Lu Y Qin Z Wang J Zheng X Lu J Zhang X. Epstein-barr virus miR-BART6-3p inhibits the RIG-I pathway. J Innate Immun (2017) 9:574–86. doi: 10.1159/000479749 28877527

[190] Rehwinkel J Gack MU. RIG-I-like receptors: their regulation and roles in RNA sensing. Nat Rev Immunol (2020) 20:537–51. doi: 10.1038/s41577-020-0288-3 PMC7094958 32203325

[191] Ge J Wang J Xiong F Jiang X Zhu K Wang Y. Epstein-barr virus-encoded circular RNA circBART2.2 promotes immune escape of nasopharyngeal carcinoma by regulating PD-L1. Cancer Res (2021) 81:5074–88. doi: 10.1158/0008-5472.CAN-20-4321 PMC8974435 34321242

[192] Farhood B Najafi M Mortezaee K. CD8(+) cytotoxic T lymphocytes in cancer immunotherapy: A review. J Cell Physiol (2019) 234:8509–21. doi: 10.1002/jcp.27782 30520029

[193] Gong W Donnelly CR Heath BR Bellile E Donnelly LA Taner HF. Cancer-specific type-I interferon receptor signaling promotes cancer stemness and effector CD8+ T-cell exhaustion. Oncoimmunology (2021) 10:1997385. doi: 10.1080/2162402X.2021.1997385 34858725 PMC8632299

[194] Lu C Klement JD Ibrahim ML Xiao W Redd PS Nayak-Kapoor A. Type I interferon suppresses tumor growth through activating the STAT3-granzyme B pathway in tumor-infiltrating cytotoxic T lymphocytes. J Immunother Cancer (2019) 7:157. doi: 10.1186/s40425-019-0635-8 31228946 PMC6589175

[195] Saleiro D Platanias LC. Interferon signaling in cancer. Non-canonical pathways and control of intracellular immune checkpoints. Semin Immunol (2019) 43:101299. doi: 10.1016/j.smim.2019.101299 31771762 PMC8177745

[196] Jorgovanovic D Song M Wang L Zhang Y. Roles of IFN-gamma in tumor progression and regression: a review. biomark Res (2020) 8:49. doi: 10.1186/s40364-020-00228-x 33005420 PMC7526126

[197] Boukhaled GM Harding S Brooks DG. Opposing roles of type I interferons in cancer immunity. Annu Rev Pathol (2021) 16:167–98. doi: 10.1146/annurev-pathol-031920-093932 PMC8063563 33264572

[198] Vaillant JA Qurie A. Interleukins . Available at: https://www.ncbi.nlm.nih.gov/books/NBK499840/#:~:text=Interleukins%20(IL)%20are%20a%20type,maturation%2C%20migration%2C%20and%20adhesion (Accessed 8/09/2023).

[199] Briukhovetska D Dorr J Endres S Libby P Dinarello CA Kobold S. Interleukins in cancer: from biology to therapy. Nat Rev Cancer (2021) 21:481–99. doi: 10.1038/s41568-021-00363-z PMC8173513 34083781

[200] Boraschi D. What is IL-1 for? The functions of interleukin-1 across evolution. Front Immunol (2022) 13:872155. doi: 10.3389/fimmu.2022.872155 35464444 PMC9020223

[201] Skinner CM Ivanov NS Barr SA Chen Y Skalsky RL. An epstein-barr virus microRNA blocks interleukin-1 (IL-1) signaling by targeting IL-1 receptor 1. J Virol (2017) 91. doi: 10.1128/JVI.00530-17 PMC5640834 28794034

[202] Chong JM Sakuma K Sudo M Osawa T Ohara E Uozaki H. Interleukin-1beta expression in human gastric carcinoma with Epstein-Barr virus infection. J Virol (2002) 76:6825–31. doi: 10.1128/jvi.76.13.6825-6831.2002 PMC136266 12050395

[203] Zhang G Tsang CM Deng W Yip YL Lui VW Wong SC. Enhanced IL-6/IL-6R signaling promotes growth and Malignant properties in EBV-infected premalignant and cancerous nasopharyngeal epithelial cells. PloS One (2013) 8:e62284. doi: 10.1371/journal.pone.0062284 23658720 PMC3641047

[204] Sorensen RB Hadrup SR Svane IM Hjortso MC Thor Straten P Andersen MH. Indoleamine 2,3-dioxygenase specific, cytotoxic T cells as immune regulators. Blood (2011) 117:2200–10. doi: 10.1182/blood-2010-06-288498 PMC3062329 21079151

[205] Burassakarn A Srisathaporn S Pientong C Wongjampa W Vatanasapt P Patarapadungkit N. Exosomes-carrying Epstein-Barr virus-encoded small RNA-1 induces indoleamine 2, 3-dioxygenase expression in tumor-infiltrating macrophages of oral squamous-cell carcinomas and suppresses T-cell activity by activating RIG-I/IL-6/TNF-alpha pathway. Oral Oncol (2021) 117:105279. doi: 10.1016/j.oraloncology.2021.105279 33819809

[206] Liu WL Lin YH Xiao H Xing S Chen H Chi PD. Epstein-Barr virus infection induces indoleamine 2,3-dioxygenase expression in human monocyte-derived macrophages through p38/mitogen-activated protein kinase and NF-kappaB pathways: impairment in T cell functions. J Virol (2014) 88:6660–71. doi: 10.1128/JVI.03678-13 PMC4054364 24696473

[207] Mikkelsen SS Jensen SB Chiliveru S Melchjorsen J Julkunen I Gaestel M. RIG-I-mediated activation of p38 MAPK is essential for viral induction of interferon and activation of dendritic cells: dependence on TRAF2 and TAK1. J Biol Chem (2009) 284:10774–82. doi: 10.1074/jbc.M807272200 PMC2667765 19224920

[208] Nicolae A Pittaluga S Abdullah S Steinberg SM Pham TA Davies-Hill T. EBV-positive large B-cell lymphomas in young patients: a nodal lymphoma with evidence for a tolerogenic immune environment. Blood (2015) 126:863–72. doi: 10.1182/blood-2015-02-630632 PMC4536540 25999451

[209] Atri-Schuller A Abushukair H Cavalcante L Hentzen S Saeed A Saeed A. Tumor molecular and microenvironment characteristics in EBV-associated Malignancies as potential therapeutic targets: focus on gastric cancer. Curr Issues Mol Biol (2022) 44:5756–67. doi: 10.3390/cimb44110390 PMC9689242 36421674

[210] Pan Y Yu Y Wang X Zhang T. Tumor-associated macrophages in tumor immunity. Front Immunol (2020) 11:583084. doi: 10.3389/fimmu.2020.583084 33365025 PMC7751482

[211] Xu Y Zeng H Jin K Liu Z Zhu Y Xu L. Immunosuppressive tumor-associated macrophages expressing interlukin-10 conferred poor prognosis and therapeutic vulnerability in patients with muscle-invasive bladder cancer. J Immunother Cancer (2022) 10. doi: 10.1136/jitc-2021-003416 PMC8961180 35338085

[212] Information, N.C.f.B. IL10 interluekin 10 [Homo sapiens (human)] . Available at: https://www.ncbi.nlm.nih.gov/gene/3586 (Accessed 08/10/2023).

[213] Liu Q Yang C Wang S Shi D Wei C Song J. Wnt5a-induced M2 polarization of tumor-associated macrophages via IL-10 promotes colorectal cancer progression. Cell Commun Signal (2020) 18:51. doi: 10.1186/s12964-020-00557-2 32228612 PMC7106599

[214] Zhang H Li R Cao Y Gu Y Lin C Liu X. Poor clinical outcomes and immunoevasive contexture in intratumoral IL-10-producing macrophages enriched gastric cancer patients. Ann Surg (2022) 275:e626–35. doi: 10.1097/SLA.0000000000004037 32541216

[215] Gao L Han H Wang H Cao L Feng WH. IL-10 knockdown with siRNA enhances the efficacy of Doxorubicin chemotherapy in EBV-positive tumors by inducing lytic cycle via PI3K/p38 MAPK/NF-kB pathway. Cancer Lett (2019) 462:12–22. doi: 10.1016/j.canlet.2019.07.016 31352079

[216] Forconi CS Mulama DH Saikumar Lakshmi P Foley J Otieno JA Kurtis JD. Interplay between IL-10, IFN-gamma, IL-17A and PD-1 expressing EBNA1-specific CD4(+) and CD8(+) T cell responses in the etiologic pathway to endemic burkitt lymphoma. Cancers (Basel) (2021) 13. doi: 10.3390/cancers13215375 PMC8582526 34771539

[217] Ren Y Yang J Li M Huang N Chen Y Wu X. Viral IL-10 promotes cell proliferation and cell cycle progression via JAK2/STAT3 signaling pathway in nasopharyngeal carcinoma cells. Biotechnol Appl Biochem (2020) 67:929–38. doi: 10.1002/bab.1856 31737947

[218] Deng G Song X Greene MI. FoxP3 in T(reg) cell biology: a molecular and structural perspective. Clin Exp Immunol (2020) 199:255–62. doi: 10.1111/cei.13357 PMC7008219 31386175

[219] Jorapur A Marshall LA Jacobson S Xu M MArubayashi S Zibinsky M. EBV+ tumors exploit tumor cell-intrinsic and -extrinsic mechanisms to produce regulatory T cell-recruiting chemokines CCL17 and CCL22. PloS Pathog (2022) 18:e1010200. doi: 10.1371/journal.ppat.1010200 35025968 PMC8791514

[220] Sausen DG Reed KM Bhutta MS Gallo ES Borenstein R. Evasion of the host immune response by betaherpesviruses. Int J Mol Sci (2021) 22. doi: 10.3390/ijms22147503 PMC8306455 34299120

[221] Zhang Y Chen Y Li Y Huang F Luo B Yuan Y. The ORF8 protein of SARS-CoV-2 mediates immune evasion through down-regulating MHC-Iota. Proc Natl Acad Sci U.S.A. (2021) 118. doi: 10.1073/pnas.2024202118 PMC8201919 34021074

[222] Kim IJ Lee YH Khalid MM Chen IP Zhang Y Ott M. SARS-CoV-2 protein ORF8 limits expression levels of Spike antigen and facilitates immune evasion of infected host cells. J Biol Chem (2023) 299:104955. doi: 10.1016/j.jbc.2023.104955 37354973 PMC10289268

[223] Becar M Kasi A. Physiology, MHC Class I. In: StatPearls. Treasure Island (FL: StatPearls Publishing (2023). 32310482

[224] Zuo J Currin A Griffin BD Shannon-Lowe C Thomas WA Ressing ME. The Epstein-Barr virus G-protein-coupled receptor contributes to immune evasion by targeting MHC class I molecules for degradation. PloS Pathog (2009) 5:e1000255. doi: 10.1371/journal.ppat.1000255 19119421 PMC2603334

[225] Griffin BD Gram AM Mulder A Van Leeuwen D Claas FH Wang F. EBV BILF1 evolved to downregulate cell surface display of a wide range of HLA class I molecules through their cytoplasmic tail. J Immunol (2013) 190:1672–84. doi: 10.4049/jimmunol.1102462 PMC3565383 23315076

[226] Baranwal AK Mehra NK. Major histocompatibility complex class I chain-related A (MICA) molecules: relevance in solid organ transplantation. Front Immunol (2017) 8:182. doi: 10.3389/fimmu.2017.00182 28293239 PMC5329007

[227] Wong TS Chen S Zhang MJ Chan JY Gao W. Epstein-Barr virus-encoded microRNA BART7 downregulates major histocompatibility complex class I chain-related peptide A and reduces the cytotoxicity of natural killer cells to nasopharyngeal carcinoma. Oncol Lett (2018) 16:2887–92. doi: 10.3892/ol.2018.9041 PMC6096257 30127876

[228] Lee SP Brooks JM Al-Jarrah H Thomas WA Haigh TA Taylor GS. CD8 T cell recognition of endogenously expressed epstein-barr virus nuclear antigen 1. J Exp Med (2004) 199:1409–20. doi: 10.1084/jem.20040121 PMC2211813 15148339

[229] Yin Y Manoury B Fahraeus R. Self-inhibition of synthesis and antigen presentation by Epstein-Barr virus-encoded EBNA1. Science (2003) 301:1371–4. doi: 10.1126/science.1088902 12958359

[230] Lista MJ Martins RP Billant O Contesse MA Findakly S Pochard P. Nucleolin directly mediates Epstein-Barr virus immune evasion through binding to G-quadruplexes of EBNA1 mRNA. Nat Commun (2017) 8:16043. doi: 10.1038/ncomms16043 28685753 PMC5504353

[231] Angrand G Quillevere A Loaec N Dinh VT Le Senechal R Chennoufi R. Type I arginine methyltransferases are intervention points to unveil the oncogenic Epstein-Barr virus to the immune system. Nucleic Acids Res (2022) 50:11799–819. doi: 10.1093/nar/gkac915 PMC9723642 36350639

[232] Ghasemi F Gameiro SF Tessier TM Maciver AH Mymryk JS. High levels of class I major histocompatibility complex mRNA are present in epstein-barr virus-associated gastric adenocarcinomas. Cells (2020) 9. doi: 10.3390/cells9020499 PMC7072773 32098275

[233] Sengupta S den Boon JA Chen IH Newton MA Dahl DB Chen M. Genome-wide expression profiling reveals EBV-associated inhibition of MHC class I expression in nasopharyngeal carcinoma. Cancer Res (2006) 66:7999–8006. doi: 10.1158/0008-5472.CAN-05-4399 16912175

[234] Tudor CS Dawson CW Eckhardt J Niedobitek G Buttner AC Seliger B. c-Myc and EBV-LMP1: two opposing regulators of the HLA class I antigen presentation machinery in epithelial cells. Br J Cancer (2012) 106:1980–8. doi: 10.1038/bjc.2012.197 PMC3388564 22588558

[235] Kamal S Kerndt CC Lappin SL. Genetics, Histocompatibility Antigen. In: StatPearls. Treasure Island (FL: StatPearls Publishing (2023). 31082067

[236] Su C Lu F Soldan SS Lamontagne RJ Tang HY Napoletani G. EBNA2 driven enhancer switching at the CIITA-DEXI locus suppresses HLA class II gene expression during EBV infection of B-lymphocytes. PloS Pathog (2021) 17:e1009834. doi: 10.1371/journal.ppat.1009834 34352044 PMC8370649

[237] Leon MaChado JA Steimle V. The MHC class II transactivator CIITA: not (Quite) the odd-one-out anymore among NLR proteins. Int J Mol Sci (2021) 22. doi: 10.3390/ijms22031074 PMC7866136 33499042

[238] Jiang XN Yu BH Yan WH Lee J Zhou XY Li XQ. Epstein-Barr virus-positive diffuse large B-cell lymphoma features disrupted antigen capture/presentation and hijacked T-cell suppression. Oncoimmunology (2020) 9:1683346. doi: 10.1080/2162402X.2019.1683346 32002294 PMC6959427

[239] Ghasemi F Tessier TM Gameiro SF Maciver AH Cecchini MJ Mymryk JS. High MHC-II expression in Epstein-Barr virus-associated gastric cancers suggests that tumor cells serve an important role in antigen presentation. Sci Rep (2020) 10:14786. doi: 10.1038/s41598-020-71775-4 32901107 PMC7479113

[240] Liu J Chen Z Li Y Zhao W Wu J Zhang Z. PD-1/PD-L1 checkpoint inhibitors in tumor immunotherapy. Front Pharmacol (2021) 12:731798. doi: 10.3389/fphar.2021.731798 34539412 PMC8440961

[241] Wang FH Wei XL Feng J Li Q Xu N Hu XC. Efficacy, safety, and correlative biomarkers of toripalimab in previously treated recurrent or metastatic nasopharyngeal carcinoma: A phase II clinical trial (POLARIS-02). J Clin Oncol (2021) 39:704–12. doi: 10.1200/JCO.20.02712 PMC8078488 33492986

[242] Guo L Zhang H Chen B. Nivolumab as programmed death-1 (PD-1) inhibitor for targeted immunotherapy in tumor. J Cancer (2017) 8:410–6. doi: 10.7150/jca.17144 PMC5332892 28261342

[243] Lim DW Kao HF Suteja L Li CH Quah HS Tan DS. Clinical efficacy and biomarker analysis of dual PD-1/CTLA-4 blockade in recurrent/metastatic EBV-associated nasopharyngeal carcinoma. Nat Commun (2023) 14:2781. doi: 10.1038/s41467-023-38407-7 37188668 PMC10184620

[244] Saad P Kasi A. Ipilimumab. Treasure Island: StatPearls Publishing (2023). 32491727

[245] Wei XL Liu QW Liu FR Yuan SS Li XF Li JN. The clinicopathological significance and predictive value for immunotherapy of programmed death ligand-1 expression in Epstein-Barr virus-associated gastric cancer. Oncoimmunology (2021) 10:1938381. doi: 10.1080/2162402X.2021.1938381 34235004 PMC8216206

[246] Quan L Chen X Liu A Zhang Y Guo X Yan S. PD-1 blockade can restore functions of T-cells in epstein-barr virus-positive diffuse large B-cell lymphoma. In Vitro. PloS One (2015) 10:e0136476. doi: 10.1371/journal.pone.0136476 26361042 PMC4567291

[247] Yilmaz E Lakhotia R Pittaluga S Muppidi J Phelan J Evans S. Phase 2 study of nivolumab in epstein-barr virus (EBV)-positive lymphoproliferative disorders and EBV-positive non-hodgkin lymphomas. Blood (2021) 138:4504. doi: 10.1182/blood-2021-151824

[248] Morrison VA. Frontline therapy with R-CHOP for diffuse large B-cell lymphoma: Where have we come (or not come)? A Perspective. J Geriatr Oncol (2021) 12:320–5. doi: 10.1016/j.jgo.2020.09.015 32972884

[249] Cui X Snapper CM. Epstein barr virus: development of vaccines and immune cell therapy for EBV-associated diseases. Front Immunol (2021) 12:734471. doi: 10.3389/fimmu.2021.734471 34691042 PMC8532523

[250] Sinha D Srihari S Beckett K Le Texier L Solomon M Panikkar A. ‘Off-the-shelf’ allogeneic antigen-specific adoptive T-cell therapy for the treatment of multiple EBV-associated Malignancies. J Immunother Cancer (2021) 9. doi: 10.1136/jitc-2020-001608 PMC7887372 33589524

[251] Prockop S Doubrovina E Suser S Heller G Barker J Dahi P. Off-the-shelf EBV-specific T cell immunotherapy for rituximab-refractory EBV-associated lymphoma following transplantation. J Clin Invest (2020) 130:733–47. doi: 10.1172/JCI121127 PMC6994129 31689242

[252] Kjeldsen JW Lorentzen CL Martinenaite E Ellebaek E Donia M Holmstroem RB. A phase 1/2 trial of an immune-modulatory vaccine against IDO/PD-L1 in combination with nivolumab in metastatic melanoma. Nat Med (2021) 27:2212–23. doi: 10.1038/s41591-021-01544-x PMC8904254 34887574

[253] Nayak-Kapoor A Hao Z Sadek R Dobbins R Marshall L Vahanian NN. Phase Ia study of the indoleamine 2,3-dioxygenase 1 (IDO1) inhibitor navoximod (GDC-0919) in patients with recurrent advanced solid tumors. J Immunother Cancer (2018) 6:61. doi: 10.1186/s40425-018-0351-9 29921320 PMC6009946

[254] Ruhl J Citterio C Engelmann C Haigh T Dzionek A Dreyer J. Heterologous prime-boost vaccination protects against EBV antigen-expressing lymphomas. J Clin Invest (2019) 129:2071–87. doi: 10.1172/JCI125364 PMC6486346 31042161

[255] Taylor GS Jia H Harrington K Lee LW Turner J Ladell K. A recombinant modified vaccinia ankara vaccine encoding Epstein-Barr Virus (EBV) target antigens: a phase I trial in UK patients with EBV-positive cancer. Clin Cancer Res (2014) 20:5009–22. doi: 10.1158/1078-0432.CCR-14-1122-T PMC4340506 25124688

[256] Hui EP Taylor GS Jia H Ma BB Chan SL Ho R. Phase I trial of recombinant modified vaccinia ankara encoding Epstein-Barr viral tumor antigens in nasopharyngeal carcinoma patients. Cancer Res (2013) 73:1676–88. doi: 10.1158/0008-5472.CAN-12-2448 PMC6485495 23348421

[257] Bian J Niu Y Ma Y Chen F Ma NA. Review on the application of PD-1 blockade in EBV-associated nasopharyngeal carcinoma immunotherapy. Appl Bionics Biomech (2022) 2022:8537966. doi: 10.1155/2022/8537966 35126664 PMC8813251

[258] Daskalogianni C Pyndiah S Apcher S Mazars A Manoury B Ammari N. Epstein-Barr virus-encoded EBNA1 and ZEBRA: targets for therapeutic strategies against EBV-carrying cancers. J Pathol (2015) 235:334–41. doi: 10.1002/path.4431 25186125

[259] Lo AK Dawson CW Lung HL Wong KL Young LS. The therapeutic potential of targeting BARF1 in EBV-associated malignancies. Cancers (Basel) (2020) 12. doi: 10.3390/cancers12071940 PMC7409022 32708965

